# Preparation and Evaluation of 6-Gingerol Derivatives as Novel Antioxidants and Antiplatelet Agents

**DOI:** 10.3390/antiox12030744

**Published:** 2023-03-17

**Authors:** Sara H. H. Ahmed, Tímea Gonda, Orinamhe G. Agbadua, Gábor Girst, Róbert Berkecz, Norbert Kúsz, Meng-Chun Tsai, Chin-Chung Wu, György T. Balogh, Attila Hunyadi

**Affiliations:** 1 Institute of Pharmacognosy, University of Szeged, H-6720 Szeged, Hungary; 2 Institute of Pharmaceutical Analysis, University of Szeged, H-6720 Szeged, Hungary; 3 Graduate Institute of Natural Products, Kaohsiung Medical University, Kaohsiung 807, Taiwan; 4 Institute of Pharmacodynamics and Biopharmacy, University of Szeged, H-6720 Szeged, Hungary; 5 Department of Chemical and Environmental Process Engineering, Budapest University of Technology and Economics, H-1111 Budapest, Hungary; 6 Interdisciplinary Centre of Natural Products, University of Szeged, H-6720 Szeged, Hungary

**Keywords:** ginger, 6-gingerol, cardiovascular disease, antiplatelet, cyclooxygenase-1, antioxidant, pharmacokinetics, BBB-PAMPA

## Abstract

Ginger (*Zingiber officinale*) is widely used as a spice and a traditional medicine. Many bioactivities have been reported for its extracts and the isolated compounds, including cardiovascular protective effects. Different pathways were suggested to contribute to these effects, like the inhibition of platelet aggregation. In this study, we synthesised fourteen 6-gingerol derivatives, including eight new compounds, and studied their antiplatelet, COX-1 inhibitor, and antioxidant activities. In silico docking of selected compounds to *h*-COX-1 enzyme revealed favourable interactions. The investigated 6-gingerol derivatives were also characterised by in silico and experimental physicochemical and blood–brain barrier-related parameters for lead and preclinical candidate selection. 6-Shogaol (**2**) was identified as the best overall antiplatelet lead, along with compounds **3** and **11** and the new compound **17**, which require formulation to optimize their water solubility. Compound **5** was identified as the most potent antioxidant that is also promising for use in the central nervous system (CNS).

## 1. Introduction

Ginger, *Zingiber officinale* Rosc. (Zingiberaceae), is a well-known culinary plant and herbal remedy for many diseases. Ginger root extracts have been reported efficient against nausea and vomiting, diarrhoea, inflammatory conditions, metabolic syndrome, hepatotoxicity, and cardiovascular diseases [1,2,3]. The plant is rich in bioactive secondary metabolites including terpenes and phenolic compounds. The latter group consists mainly of gingerols, shogaols, paradols, and zingerone [4,5], among which 6-gingerol has been reported to be the most abundant in fresh ginger roots [6].

A plethora of preclinical studies demonstrated antioxidant [7,8], antimicrobial [9,10], anti-inflammatory [11], neuroprotective [12], antiplatelet [13], anti-obesity [14], antihepatotoxic [15], and anticancer effects for 6-gingerol [16]. Great efforts have been devoted to synthesizing and studying semisynthetic derivatives inspired by the structure of 6-gingerol. In our recent review, we have discussed such studies covering more than 150 semi-synthetic compounds, based on which the antiplatelet activity of gingerol derivatives seems to be the most promising [17].

Cardiovascular diseases (CVDs) represent a leading cause of death worldwide, with approximately 17.9 million deaths every year, according to the World Health Organisation (WHO) reports [18]. Many factors are implicated in CVDs’ development and progression, including an imbalance between haemostasis and thrombosis [19]. Platelets represent a key element in this due to their crucial role in the coagulation cascade, and antiplatelet therapies are of fundamental importance in the treatment of cardiovascular and in cerebrovascular pathologies [20]. Cyclooxygenase 1 (COX-1), constitutively expressed in all tissues, is a key regulator of platelet activation [21]. It has also been reported that high reactive oxygen species (ROS) levels contribute to platelet activation and play a causal role in CVDs [22]. While currently marketed antiplatelet drugs are undoubtedly efficient, all of them have some clinical limitations [23,24]. Aspirin is a good example of this: it significantly reduces morbidity in CVDs [25], but its gastrointestinal (GI) side effects limit its use, especially in those patients who are susceptible to, or already experience, gastrointestinal ulcers [26]. Therefore, there is still a need for additional safe and efficient alternatives.

Phytotherapy may offer complementary supportive treatment options for CVDs [27], and ginger has been suggested for this purpose [3] due to its effectiveness in reducing platelet aggregation [2,28,29,30,31]. Related clinical studies have also been conducted. Young et al. reported an increase in the antiplatelet activity upon ginger consumption by nifedipine-treated patients [32]. A similar effect was observed upon ginger administration in patients with coronary artery disease [33]. On the other hand, no effect was observed on platelet aggregation and coagulation when ginger was administered to healthy humans [34,35]. Furthermore, promising results have also been reported for the antiplatelet activity of semi-synthetic gingerol derivatives [36,37].

Ginger and many of its constituents, particularly gingerols and shogaols, are considered potent antioxidants through scavenging various biologically relevant free radicals [38] and modulating a range of redox signalling pathways [17]. It is worth mentioning that the modulation of antioxidant pathways by 6-gingerol protects H9c2 cardiomyocytes from hypoxia-induced damage [39], and that ginger constituents also inhibit the NLRP3 inflammasome [40] that has a key importance concerning cardiovascular health [41,42].

In the present work, we aimed to introduce different changes to the skeleton of 6-gingerol, and test the antiplatelet, COX-1 inhibitory, and antioxidant activity to evaluate their pharmacodynamic potential as cardio- and cerebrovascular protective agents. Furthermore, it was also our aim to characterize key physicochemical parameters and central nervous system (CNS)-related basic pharmacokinetic behaviour of the gingerol derivatives, including their in vitro blood–brain barrier permeability, to evaluate their additional potential as CNS antioxidants.

## 2. Materials and Methods

### 2.1. General Information

Reagents were purchased from Sigma (Merck KGaA, Darmstadt, Germany) unless otherwise stated. Solvents (analytical grade for synthetic work and flash chromatography purifications and high-performance liquid chromatography (HPLC)-grade for analytical and preparative HPLC work) were obtained from Macron Fine Chemicals (Avantor Performance Materials, Center Valley, PA, USA), Chem-Lab NV (Zedelgem, Belgium), VWR International S.A.S., and Fontenay-sous-Bois, France. A commercial ginger extract was purchased from Xi’an Pincredit Bio-Tech Co., Ltd., China. COX-1 kit was obtained from VWR International Kft. Debrecen, Hungary (original source: Biovision Inc., Milpitas, CA, USA).

For purification of the compounds, flash chromatography was used on a CombiFlashfi Rf+ Lumen apparatus (TELEDYNE Isco, Lincoln, NE, USA) equipped with evaporative light scattering (ELS) and diode array detectors, and the stationary phases were RediSep prefilled silica columns and RediSep cartridges (Teledyne Isco Inc., Lincoln, NE, USA). Hereinafter, solvent system compositions are always given in volumetric ratios.

Preparative purifications over RP-HPLC were performed on a Kinetex XB C18 (5 μm, 250 × 21.2 mm) column on an Armen Spot Prep II integrated HPLC purification system (Gilson, Middleton, WI, USA) with dual-wavelength detection, with an adequately chosen combination of acetonitrile–water, and a flow rate of 15 mL/min. The purity of the compounds obtained was determined by RP-HPLC analyses on a system of two Jasco PU 2080 pumps, a Jasco AS-2055 Plus intelligent sampler connected to a JASCO LC-Net II/ADC equipped with a Jasco MD-2010 Plus PDA detector (Jasco International Co. Ltd., Hachioji, Tokyo, Japan) utilizing a Kinetex C-18 (5 μm, 250 × 4.6 mm) column (Phenomenex Inc., Torrance, CA, USA) and applying a gradient of 30%–100% aqueous AcN in 30 min followed by 100% ACN for 10 min with a flow rate of 1 mL/min. Analysis of samples from the PAMPA and kinetic solubility assays was performed the same way, by using 3-point calibrations and integrating each compound at its UV absorption maximum.

^1^H- and ^13^C NMR spectra were recorded in CDCl_3_ or CD_3_OD using 5 mm tubes at room temperature on a Bruker DRX-500 spectrometer at 500 (^1^H) and 125 (^13^C) MHz with the deuterated solvents’ signal taken as reference. The heteronuclear single quantum coherence (HSQC), heteronuclear multiple bond correlation (HMBC), ^1^H-^1^H correlation spectroscopy (COSY), and nuclear Overhauser effect spectroscopy (NOESY) spectra were obtained using the standard Bruker pulse programs. ^1^H- and ^13^C NMR spectra for compounds **4**, **12**, **14**–**18** and **22** are available in the Supplementary Materials, Figures S9–S24. High resolution mass spectroscopy (HRMS) spectra were recorded on a Q-Exactive Plus hybrid quadrupole-orbitrap mass spectrometer (Thermo Scientific, Waltham, MA, USA) equipped with heated electrospray ionisation (HESI-II) probe that was used in positive or negative mode per needed. HRMS spectra for compounds **4**, **12**, **14**–**18** and **22** are available as Supplementary Materials, Figures S1–S8.

All bioactivity data processing, including the calculation of inhibition percentage, mean and corresponding standard error of the mean (SEM), and IC_50_ values, was performed by GraphPad Prism 8.0 (La Jolla, CA, USA). IC_50_ values were calculated from the sigmoidal dose–response curves obtained by the log(inhibitor) vs. response and variable slope (DPPH assay) or the log(inhibitor) vs. normalised response and variable slope nonlinear regression model. On the results obtained, no statistical evaluation was performed; instead, differences greater than two-fold were considered relevant. Plotting of the antiplatelet and COX-1 inhibitory IC_50_ values and the linear regression of the data was performed by Microsoft Excel.

### 2.2. Isolation and Semi-synthesis

#### 2.2.1. Purification of 6-gingerol ((*S*)-5-hydroxy-1-(4-hydroxy-3-methoxyphenyl)decan-3-one) (**1**)

Ginger extract was purchased from Xi’an Pincredit Bio-Tech Co., Ltd., Xi’an, China. 6-Gingerol (**1**) was purified from the crude extract at up to a 4 g scale using flash chromatography (Silica, gradient elution of 0–10% of acetone in *n*-hexane) and obtained as a dark yellow oil (36.8% yield). 6-Gingerol (**1**) was then utilised to synthesize 6-shogaol (**2**), and subsequently 4,5-dihydro-6-shogaol (**3**), as published previously [43]. Compound **11** was derived from vanillin (**7**) and 2,4-nonanedione (**10**), and compound **13** from compound **11**, as published previously [44].

#### 2.2.2. Synthesis of 1-(4-hydroxy-3-methoxyphenyl)decan-3-one oxime (**4**)

Hydroxylamine hydrochloride (228 mg, 3.3 mmol) was added to a solution of compound **3** (304 mg, 1.1 mmol) in MeOH (5 mL) at room temperature. The reaction was monitored by thin-layer chromatography (TLC), the reaction mixture was purified by flash chromatography (Silica, gradient elution of 0–30% of EtOAc in *n*-hexane) to afford compound **4**, a pale-yellow solid (255 mg, 74.8%) [37].

Compound **4**. HRESIMS: C_17_H_27_NO_3_, [M+H]^+^ *m/z* = 294.20694 (calcd 294.20692), ^1^H NMR (500 MHz, in CDCl_3_): δ_H_ 6.83 (m, 1H), 6.70 (dd, 2H, *J* = 13.8, 7.6 Hz), 3.88 (d, 3H, *J* = 3.5 Hz), 3.48 (s, 1H), 2.77 (q, 2H, *J* = 8.0 Hz), 2.60 (m, 1H), 2.46 (m, 1H), 2.36 (m, 1H), 2.10 (t, 1H, *J* = 7.6 Hz), 1.50 (m, 2H), 1.31 (m, 2H), 1.28 (m, 7H), 0.88 (td, 3H, *J* = 7.0, 2.9 Hz) ppm. ^13^C NMR (125 MHz, in CDCl_3_): δ_C_ 161.8, 161.7, 146.6, 144.1, 144.0, 133.6, 133.4, 121.0, 114.5, 111.1, 56.1, 36.4, 34.6, 32.5, 31. 9, 31.9, 31.5, 30.0, 30.0, 29.4, 29.2, 27.9, 26.4, 25.8, 22.8, 14.2.

#### 2.2.3. Synthesis of (3*R*,5*S*)-1-(4-hydroxy-3-methoxyphenyl)decane-3,5-diol (**5**) and (3*S*,5*S*)-1-(4-hydroxy-3-methoxy phenyl)decane-3,5-diol (**6**)

Compound **1** (100 mg, 0.34 mmol) was dissolved in EtOH (10 mL) and NaBH_4_ was added (38 mg, 1 mmol). The reaction was monitored by means of TLC and after completion (1 h) the solvent was evaporated in vacuo and water (20 mL) was added to the residue. The aqueous phase was extracted with EtOAc (3 × 20 mL), and the combined organic phase was dried over Na_2_SO_4_, filtered, and evaporated in vacuo resulting in a yellow oil (85 mg, 84%), which contained the gingerdiols in high purity (>95%), which were separated via preparative HPLC (50% aqueous AcN) to yield compound **5** as a faint yellow oil, and compound **6** as a white powder. The compounds’ MS and NMR spectra were in good agreement with the literature data [38].

#### 2.2.4. Synthesis of (*E*)-1-(4-hydroxyphenyl)dec-1-ene-3,5-dione (**12**)

*p*-Hydroxybenzaldehyde **8** (1000 mg, 8.2 mmol), boron trioxide (2280 mg, 32.8 mmol), and 2,4-nonanedione (2800 mg, 24.6 mmol) were mixed with 2 mL of DMF and heated to 90 °C. A solution of isobutylamine (150 mg = 202 µL, 4 mmol) in 2 mL of DMF was added dropwise over 2 h. The reaction mixture was stirred at 90 °C for 1 h then 50 mL of water was added. The resulting mixture was stirred at 60 °C for 1 h at room temperature overnight. The reaction mixture was extracted with EtOAc and the solvent was evaporated. Purification by flash chromatography (Silica, gradient elution, 5–10% EtOAc in *n*-hexane) afforded compound **12**, a yellow solid (179 mg, 8.5%) [39].

Compound **12**. HRESIMS: C_16_H_20_O_3_, [M−H]^−^ *m/z* = 259.1332, (calcd 260.13342), ^1^H NMR (500 MHz, in CD_3_OD): δ_H_ 7.57 (d, 1H, *J* = 15.9 Hz), 7.50 (d, 2H, *J* = 8.6 Hz), 6.84 (m, 2H), 6.51 (d, 1H, *J* = 15.9 Hz), 2.42 (m, 2H), 1.67 (p, 2H, *J* = 7.7, 7.3 Hz), 1.39 (m, 4H), 0.97 (t, 3H, *J* = 7.0 Hz) ppm. ^13^C NMR (125 MHz, in CD_3_OD): δ_C_ 201.6, 179.6, 160.9, 141.3, 2 × 131.0, 128.1, 120.6, 2 ×116.9, 44.3, 40.9, 32.6, 26.5, 23.5, 14.3.

#### 2.2.5. Synthesis of 2-methoxy-4-(2-(3-pentyl-1H-pyrazol-5-yl)ethyl)phenol (14), 4-(2-(3-pentyl-1H-pyrazol-5-yl)ethyl)phenol (**15**), and (E)-4-(2-(3-pentyl-1H-pyrazol-5-yl)vinyl)phenol (**16**)

To a stirred solution of compounds **11** or **12** (164 mg, 0.57 mmol/100 mg, 0. 38 mmol) in ethanol (2 mL), hydrazine monohydrate (71 mg = 71 µL, 1.4 mmol/134 mg = 48 µL, 2.67 mmol) and a catalytic amount of concentrated HCl were added. The reaction mixture was refluxed for 6 h, cooled to ambient temperature, evaporated in vacuo, and purified using preparative HPLC (MeCN: H_2_O 40:60 for compound **14** and 42:58 for **15** and **16**) to afford compounds **14** (270 mg, 17.8%), **15**, (22 mg, 22.3%) or **16**, (23 mg, 23.6%) as pale-yellow solids [40].

Compound **14**. HRESIMS: C_17_H_24_N_2_O_2_, [M+H]^+^ *m/z* = 289.19111 (calcd 289.19160). ^1^H NMR (CDCl_3_, 500 MHz): δ_H_ 6.83 (d, 1H, *J* = 8.0 Hz), 6.70 (dd, 1H, *J* = 8.0, 2.0 Hz), 6.67 (d, 1H, *J* = 2.0 Hz), 5.84 (s, 1H), 3.84 (s, 3H), 3.48 (s, 1H), 2.89 (m, 4H), 2.59 (t, 2H, *J* = 7.7 Hz), 1.64 (m, 2H), 1.35 (m, 2H), 1.33 (m, 2H), 0.90 (t, 3H, *J* = 7.1 Hz) ppm. ^13^C NMR (125 MHz, in CDCl_3_): δ_C_ 146.6, 144.2, 133.6, 121.2, 114.5, 111.3, 102.6, 56.1, 35.6, 31.7, 29.6, 29.2, 27.0, 22.6, 14.1.

Compound **15**. HRESIMS: C_16_H_22_N_2_O, [M+H]^+^ *m/z* = 259.18057 (calcd 259.18104), ^1^H NMR (500 MHz, in CDCl_3_): δ_H_ 6.99 (d, 2H, *J* = 8.4 Hz), 6.71 (d, 2H, *J* = 8.5 Hz), 5.86 (s, 1H), 3.48 (s, 1H), 2.88 (t, 2H, *J* = 3.9 Hz), 2.87 (t, 2H, *J* = 4.2 Hz), 2.59 (t, 2H, *J* = 7.7 Hz), 1.63 (m, 2H), 1.34 (m, 2H), 1.31 (t, 2H, *J* = 3.9 Hz), 0.90 (t, 3H, *J* = 7.1 Hz) ppm. ^13^C NMR (125 MHz, in CDCl_3_): δ_C_ 154.7, 149.4, 148.9, 133.1, 2 × 129.6, 2 × 115.6, 102.6, 34.9, 31.6, 29.3, 29.1, 27.1, 22.6, 14.1.

Compound **16**. HRESIMS: C_16_H_20_N_2_O, [M+H]^+^ *m/z* = 257.16489 (calcd 257.16539), ^1^H NMR (500 MHz, in CD_3_OD): δ_H_ 7.34 (d, 2H, *J* = 8.6 Hz), 7.01 (d, 1H, *J* = 16.5 Hz), 6.85 (d, 1H, *J* = 16.5 Hz), 6.77 (d, 2H, *J* = 8.6Hz), 6.24 (s, 1H), 2.62 (t, 2H, *J* = 7.6 Hz), 1.67 (m, 2H), 1.38 (m, 2H), 1.36 (m, 2H), 0.92 (t, 3H, *J* = 6.8 Hz) ppm. ^13^C NMR (125 MHz, in CD_3_OD): δ_C_ 158.6, 131.1, 130.2, 2 × 128.8, 2 × 116.6, 103.8, 32.6, 30.3, 23.4, 14.3.

#### 2.2.6. Synthesis of (*E*)-2-methoxy-4-(2-(3-pentylisoxazol-5-yl)vinyl)phenol (**17**) and (*E*)-4-(2-(3-pentylisoxazol-5-yl)vinyl)phenol (**18**)

Hydroxylamine hydrochloride (144 mg, 2.1 mmol/134 mg, 1.9 mmol) and pyridine (165 mg = 169 µL, 2.1 mmol/152 mg = 155 µL, 1.9 mmol) were added to a stirred solution of compounds **11** and **12** separately (122 mg, 0.42 mmol/100 mg, 0.38 mmol) in ethanol (4 mL) and refluxed for 6 hrs. The reaction was monitored by TLC. Subsequently, the mixture was cooled to ambient temperature, evaporated in vacuo, and purified by flash chromatography (Silica, gradient elution, 0–20% acetone in *n*-hexane) to afford compound **17**, a pale-yellow solid (96 mg, 79.3%), while product **18** was obtained as a pure compound from the reaction mixture as a pale-yellow solid (98 mg, 99%) [40].

Compound **17**. HRESIMS: C_17_H_21_NO_3_, [M+H]^+^ *m/z* = 288.15999 (calcd 288.15996), ^1^H NMR (500 MHz, in CDCl_3_): δ_H_ 7.22 (d, 1H, *J* = 16.4 Hz), 7.04 (dd, 1H, *J* = 8.1, 2.0 Hz), 7.01 (d, 1H, *J* = 2.0 Hz), 6.92 (d, 1H, *J* = 8.1 Hz), 6.77 (d, 1H, *J* = 16.4 Hz), 6.06 (s, 1H), 5.77 (s, 1H), 3.95 (s, 3H), 2.66 (t, 2H, *J* = 7.5 Hz), 1.68 (m, 2H), 1.37 (m, 2H), 1.36 (m, 2H), 0.91 (t, 3H, *J* = 7.2 Hz) ppm. ^13^C NMR (125 MHz, in CDCl_3_): δ_C_ 168.5, 164.6, 147.0, 134.6, 128.5, 121.5, 114.9, 111.3, 108.9, 100.6, 56.1, 31.5, 28.2, 26.2, 22.5, 14.1.

Compound **18**. HRESIMS: C_16_H_19_NO_2_, [M−H]^−^ *m/z* = 256.13644 (calcd 256.13375). ^1^H NMR (500 MHz, in CDCl_3_): δ_H_ 7.39 (dd, 2H, *J* = 8.6, 1.8 Hz), 7.23 (d, 1H, *J* = 16.4 Hz), 6.85 (dt, 2H, *J* = 8.6, 2.9, 2.1 Hz), 6.77 (d, 1H, *J* = 16.4 Hz), 6.06 (s, 1H), 3.49 (s, 1H), 2.66 (t, 2H, *J* = 7.5 Hz), 1.68 (m, 2H), 1.36 (m, 2H), 1.35 (m, 2H), 0.90 (t, 3H, *J* = 7.2 Hz) ppm. ^13^C NMR (125 MHz, in CDCl_3_): δ_C_ 168.7, 164.6, 157.1, 134.4, 2 × 128.8, 128.6, 2 × 116.0, 111.2, 100.6, 31.5, 28.2, 26.2, 22.5, 14.0.

#### 2.2.7. Synthesis of *N*-heptyl-3-(4-hydroxyphenyl) propenamide (**22**)

A solution of compound **21** (300 mg, 1.5 mmol) in dry CH_2_Cl_2_ (10 mL) was cooled to 0 °C, DCC (316 mg) and DMAP (18 mg, 0.2 mmol) were added, and the mixture was stirred for 1 h at 0° C, then heptylamine (153 mg = 0.197 mL) was added and the reaction was monitored over TLC. Subsequently, the solvent was evaporated, redissolved in dichloromethane, washed with NaHCO_3_ solution, and the organic phase was evaporated to dryness. The product was purified by flash chromatography (Silica, gradient elution of 20-30% acetone in *n*-hexane) to yield compound **22** as a white solid (431 mg, 92.7%) [41].

Compound **22**. HRESIMS: C_17_H_27_NO_3_, [M+H]^+^ *m/z* = 294.20680, (calcd 294.20692), ^1^H NMR (500 MHz, in CDCl_3_): δ_H_ 6.83 (d, 1H, *J* = 8.1 Hz), 6.72 (d, 1H, *J* = 2.0 Hz), 6.68 (dd, 1H, *J* = 8.0, 2.0 Hz), 5.47 (s, 1H), 3.87 (s, 3H), 3.20 (td, 2H, *J* = 7.2, 5.7 Hz), 2.89 (t, 2H, *J* = 7.5 Hz), 2.42 (t, 2H, *J* = 7.5 Hz), 1.43 (m, 2H), 1.26 (m, 8H), 0.88 (t, 3H, *J* = 6.9 Hz) ppm. ^13^C NMR (125 MHz, in CDCl_3_): δ_C_ 172.2, 146.6, 144.2, 133.0, 121.0, 114.5, 111.2, 56.3, 39.7, 39.2, 31.9, 31.7, 29.8, 29.1, 27.0., 22.7, 14.2.

### 2.3. Biological Activity

#### 2.3.1. Antiplatelet Activity

Human platelet suspension (3 × 10^8^/mL in Tyrode’s buffer) was prepared as previously described [42]. The protocol for this study was approved by the institutional review board of Kaohsiung Medical University Hospital (Kaohsiung City, Taiwan). Platelets pre-treated with DMSO (vehicle control) or test compounds were stimulated with arachidonic acid (AA), and platelet aggregation was measured using turbidimetric aggregometer (Chrono-Log Co., Havertown, PA, USA) at 37 °C under stirring conditions (1200 rpm).

#### 2.3.2. Diphenyl-2-picrylhydrazyl (DPPH) Scavenging Activity

DPPH (1,10-diphenyl-2-picrylhydrazyl) was purchased from Thermo Fisher Scientific. DPPH free radical scavenging assay was performed with some modifications based on the method by previous study [43]. Briefly, in a 96-well microplate, microdilutions of samples (100 µL, starting from 200 µM in HPLC grade MeOH) were made and 100 µL of DPPH reagent (100 µM in MeOH) was added. After 30 min at room temperature in the dark, the absorbance was measured at 550 nm using a FluoStar Optima plate reader (software version 2.20R2, BMG Labtech Ortenberg, Germany). For the blank control, MeOH was used. The scavenging activity was calculated as Inhibition (%) = (A_0_ − A_s_)/A_0_ × 100, and IC_50_ values were calculated by GraphPad Prism 8.0 (La Jolla, CA, USA).

#### 2.3.3. Oxygen Radical Absorbance Capacity (ORAC)

AAPH ((2,20-Azobis(2-methyl-propionamidine) dihydrochloride) free radical and Trolox standard were purchased from Sigma-Aldrich Hungary. Fluorescein was purchased from Fluka Analytical, Tokyo, Japan. ORAC assay was carried out In a 96-well microplate based on the method from previous study [44]. Briefly, 20 µL of the samples (1 µM final concentration, dissolved in phosphate buffer, pH = 7.4, containing 1% MeOH) were mixed with 60 µL of AAPH (12 mM final concentration, dissolved in phosphate buffer, pH = 7.4) and 120 µL of fluorescein solution (70 nM final concentration, dissolved in phosphate buffer), then the fluorescence was measured (excitation at λ = 485 nm, and emission at λ = 520 nm) through 3 h with 1.5-min cycle intervals with a BMG Labtech FluoStar Optima plate-reader. All experiments were carried out in triplicate, and Trolox was used as standard. The antioxidant capacity is expressed as Trolox Equivalent (TE), as calculated using GraphPad Prism 8.0 (La Jolla, CA, USA).

#### 2.3.4. Xanthine Oxidase Inhibitory Activity

The xanthine oxidase (XO) inhibitory activity of the compounds was determined using continuous spectrophotometric rate based on a modified protocol of Sigma. The samples were prepared in. In a 96-well plate, the final reaction mixture consisted of 10 µL of sample (dissolved in DMSO, 30 mM stock solution, 100 µM final), 100 µL of xanthine solution (0.15 mM, pH = 7.4), 140 µL of buffer (potassium phosphate, pH = 7.5) and 50 µL of XO (0.2 units/mL). When measuring the enzyme activity, control buffer was used in place of the sample. Allopurinol was applied as a control. The reaction was initiated by the automatic addition of 50 µL of XO solution. The absorbance of XO-induced uric acid production from xanthine was measured at 290 nm for 3 min in a 96-well plate on a BMG Labtech FluoStar Optima plate reader. The inhibitory percentage values were calculated by using Graph Pad Prism 8.0 (La Jolla, CA, USA).

#### 2.3.5. Peroxynitrite Scavenging Activity

Peroxynitrite was synthesised by a continuous flow system using syringe pumps as published previously [45]. Briefly, an acidic solution of hydrogen peroxide (0.6 M H_2_O_2_, 0.7 M HCl) was pumped to a junction alongside of sodium nitrite solution (0.6 M) at a flow rate of 1.5 mL/min. After passing 10 cm of tubing, it was mixed with a sodium hydroxide solution (1.5 M), also pumped at 1.5 mL/min. The resulting peroxynitrite solution was a bright yellow colour. The tubing around the reaction was submerged in ice. The concentration of the solution was determined by spectrophotometry and was adjusted to 30 mM with 0.1 M NaOH solution.

In a 96-well microplate 245 µL of pyrogallol red (100 µM final concentration, dissolved in 0.1 M glycine buffer) was mixed with 50 µL of sample (0.5 mM final concentration, dissolved in DMSO) and 5 µL ONOO^–^ solution (500 µM final concentration, freshly prepared). After mixing and keeping it at room temperature for 30 min the absorbance was measured at 550 nm using a FluoStar Optima plate reader (software version 2.20R2, BMG Labtech, Ortenberg, Germany).

#### 2.3.6. COX-1 Inhibitory Activity

COX-1 inhibitory activity was tested based on the fluorometric method as described in BioVision’s COX-1 inhibitor screening kit leafkit (K548-100, BioVision, CA, USA). Sample solutions were prepared by dissolving in DMSO and subsequently buffer, to get desired concentrations. In a 96-well white plate (655101, F-bottom, Grenier bio-one, Germany), 80 µL reaction mix (containing 76 µL assay buffer, 1 µL COX Probe, 2 µL COX cofactor, and 1 µL COX-1 enzyme) was added to 10 µL sample solution, DMSO and assay buffer to get test wells assigned for sample screen (S), negative control (N) and blank, respectively. An aliquot of 10 µL of arachidonic/NaOH solution was added to each well using a multi-channel pipette to initiate the reaction at the same time, and the fluorescence of each well was measured kinetically at Ex/Em 550/610 nm, at 25OC for 10 min using a FluoStar Optima plate reader (BMG Labtech, Ortenberg, Germany). The COX inhibitory activity of SC560, a standard inhibitor, was also determined.

The change in fluorescence between two points, T1 and T2 were determined, and relative inhibition was calculated according to the following equation:

% Inhibition = (ΔN − ΔS)/ΔN × 100 (1)

where N is the absorbance of the negative control, and S is that of the sample.

Dose-effect studies on the compounds were used to determine the concentration that inhibits 50% of the enzyme activity. The sigmoidal dose–response model was obtained by using the software GraphPad Prism 8.0 (La Jolla, CA, USA), and these were used to determine the IC_50_ values of the compounds.

#### 2.3.7. Physicochemical Character and Blood–brain Barrier Specific Permeability

Basic physicochemical parameters for drug design and candidate selection were calculated by Percepta Software Package (ACD/Labs, Toronto, Ontario, Canada) [46]. Tautomers and it is distributions for compounds **11**, **12** and **13** were generated by Marvin Sketch and Tautomer Generator (Chemaxon Ltd., Budapest, Hungary) [47], which is freely accessible with academic license.

For kinetic aqueous solubility studies each sample was dissolved in DMSO to make 10 mM stock solutions. In a 96-well polypropylene plate (Greiner Bio-One, Kremsmünster, Austria), 15 μL stock solutions were added to 285 μL PBS (pH 7.4) to make starting donor solution with 500 μM as target concentration. For each sample, 3 replicates were measured. The samples were covered and shaken at 37 °C, 300 rpm for 2 h (Heidolph Titramax 1000, Heidolph Instruments GmbH & Co. KG, Schwabach, Germany). After that, each sample was transferred into a filter plate (MSSBLPC, Multiscreen Filter plate, Merck kGaA) and filtered (Vaccum Manifold, Merck kGaA, Darmstadt, Germany). The filtrates were transferred into HPLC vials, and acetonitrile (AcN) was added to aliquot to avoid precipitation. The final solvent ratio was AcN:PBS (70:30). Filtrate concentration was determined by HPLC-UV (see Section 2.1) using 3-points calibration.

Blood–brain barrier-specific (BBB) permeability measurements were carried out us- ing the PAMPA-BBB model. First, solutions with 500 μM target concentration were prepared as described for the kinetic solubility study. The solutions were sonicated for 10 min at room temperature. To prepare the artificial BBB-specific membrane, 16 mg BPLE were dissolved in 600 μL of *n*-dodecane:*n*-hexane (25:75). Each well of the donor plate (MultiscreenTM-IP, MAIPNTR10, pore size 0.45 mm, Merck kGaA) was coated with 5 μL lipid solution and fitted into the acceptor plate containing 300 μL PBS (pH 7.4) with 5% DMSO, and 150–150 μL of the PBS solutions (made from the DMSO stock solutions) were placed on the donor plate’s artificial membrane. The sandwich plate system was covered with a tissue of wet paper and a plastic lid to avoid evaporation of the solvent, and it was incubated at 37 °C for 4 h. In the end, the initial 500 μM solutions ($c_{D}\left(0\right)$), the donor ($c_{D}\left(t\right)$) and acceptor solution ($c_{A}\left(t\right)$) were analysed by HPLC-UV (see Section 2.1). BBB permeability was calculated using the effective permeability equation used for iso-pH conditions described by A. Avdeef [48] as follows.

(2) $$P_{e}=\frac{-2.303}{A\cdot \left(t-\tau_{ss}\right)}\cdot \left(\frac{1}{1+r_{v}}\right)\cdot \mathrm{lg}\left[-r_{v}+\left(\frac{1+r_{v}}{1-MR}\right)\cdot \frac{c_{D}\left(t\right)}{c_{D}\left(0\right)}\right]$$

(3) $$MR=1-\frac{c_{D}\left(t\right)}{c_{D}\left(0\right)}-\frac{V_{A}c_{A}\left(t\right)}{V_{D}c_{D}\left(0\right)}$$

where *A* is the filter area (0.3 cm^2^), *V_D_* and *V_A_* are the volumes in the donor (0.15 cm^3^) and acceptor phase (0.3 cm^3^), *t* is the incubation time (s), $\tau_{ss}$ is the time to reach steady state (s), $c_{D}\left(t\right)$ is the concentration of the compound in the donor phase at time point *t* (mol/cm^3^), $c_{D}\left(0\right)$ is the concentration of the compound in the donor phase at time point zero (mol/cm^3^), $c_{A}\left(t\right)$ is the concentration of the compound in the acceptor phase at time point *t* (mol/cm^3^), $r_{v}$ is the aqueous compartment volume ratio (*V_D_*/*V_A_*).

#### 2.3.8. Molecular Docking

Compounds’ structures were drawn and saved in PDB format using ChemDraw 12.0.2 software (ACD/LABS, Advanced Chemistry Development, Inc.) [45]. The *h*-COX-1 enzyme structure was retrieved from the PDB database. PDB files for the enzyme and compounds were converted to the PDBQT format using the graphical user interface of AutoDock4 (The Scripps Research Institute). A grid box (X: −33.050, Y: −47.920, and Z: 0.234; the number of grid points in the three dimensions [npts]: X: 40, Y: 60, and Z: 60; spacing: 0.375) was set to include the amino acids mentioned by L. Tóth to characterize the binding site [46]. Docking parameters were set to the default values and ligands were docked via the Lamarckian algorithm. The binding energies were obtained from the resulting DLG files, and interactions visualisation was achieved via Biovia (Discovery Studio visualizer version 21.1.0.20298; Dassault Systèmes, Vélizy-Villacoublay, France) after conversion of the docked PDBQT files into PDB files using OpenBabel GUI software version 2.4.1 [47].

## 3. Results and Discussion

### 3.1. Chemistry

In this work, a commercially available ginger extract was utilised to obtain significant amounts of our selected starting material, 6-gingerol (**1**), which could be obtained in a single-step purification using flash chromatography. Using compound **1** as a starting material, 6-Shogaol (**2**) was synthesised according to a literature method [43], and subsequent hydrogenation resulted in 6-paradol (**3**) [43] in an excellent yield. The reaction of **3** with hydroxylamine hydrochloride resulted in an oxime derivative as a mixture of (*E*/*Z*) isomers (**4**).

**Scheme 1:**
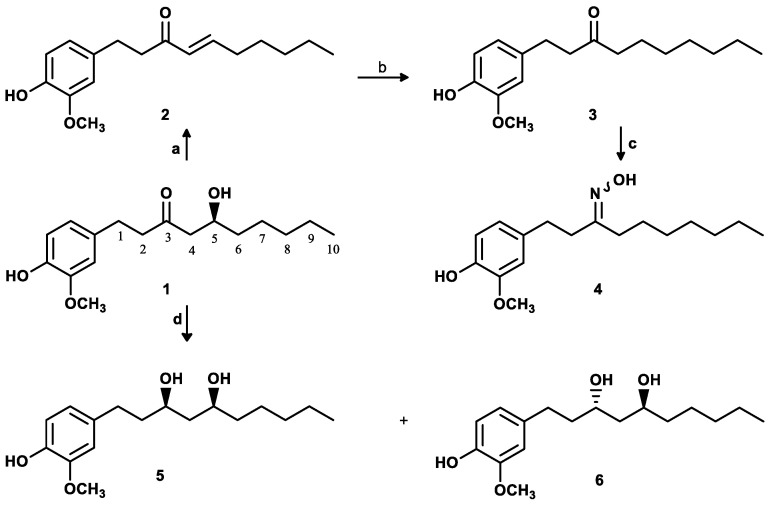
Semi-synthesis of compounds **2**–**6** from 6-gingerol (**1**). Reaction conditions: **a**. *p*TsOH/toluene/110 °C; **b**. H_2_/Pd/C/EtOAc/r.t.; **c**. NH_2_OH.HCl/EtOH/rt; **d**. NaBH_4_/MeOH.

Reduction of the keto function of 6-gingerol resulted in 6-gingerdiol epimers **5** and **6** in a ratio of 3 to 2, respectively. The compounds were separated by preparative HPLC, and their structures were confirmed by HRMS and one- and two-dimensional NMR techniques, and by comparing their relevant spectral data to literature values [49,50,51]. Heating and cooling did not affect the stereoselectivity of the reaction. Compounds prepared directly from 6-gingerol are presented in Scheme 1.

**Scheme 2:**
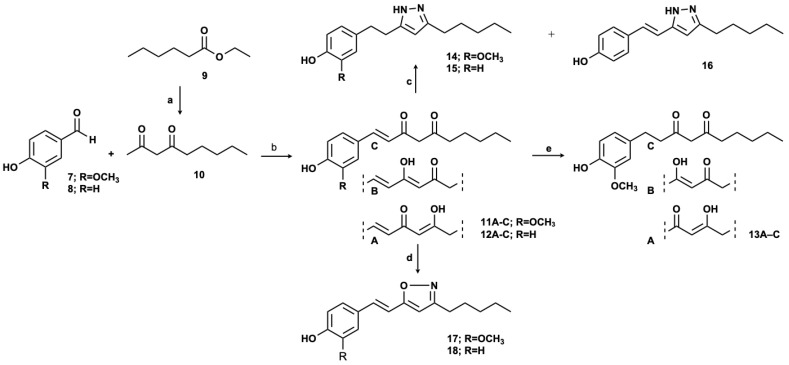
Preparation of gingerdione derivatives **11**–**13** and their heterocyclic analogues **14**–**18**. Reaction conditions: **a**. 1. NaH/Et_2_O/Acetone/0 °C, 2. EtOH/HCl; **b**. B_2_O_3_/*i*BuNH_2_/DMF/90 °C; **c**. H_6_N_2_O/HCl/EtOH/80 °C; **d**. NH_2_OH.HCl/pyridine/ethanol/80 °C; **e**. H_2_/Pd/C/EtOAc/rt. Major tautomers of compounds **11**–**13** are indicated with A, B, and C.

We also aimed to expand our study towards the chemical space around 6-dehydrogingerdione. To achieve this, a total synthetic approach was adopted (Scheme 2). First, 6-dehydrogingerdione (**11**) and its analogue lacking the aromatic methoxy function were synthesised starting from vanillin (**7**) or 4-hydroxybenzaldehyde (**8**) and 9-nonanedione according to a published method [44]. Catalytic hydrogenation of the double bond yielded compound **13** (6-gingerdione) [44]. The structure of compound **12** was confirmed using HRMS and NMR, and although the proton resonance of the enol methylene was not observed, the presence of carbon atoms at δ_C_ 201.6 and 179.6 clearly showed that **12** undergoes keto-enol tautomerisation in solution.

To facilitate exploring structure–activity relationships, oxygen and nitrogen-containing heterocyclic analogues were also prepared. Interestingly, the reaction of dehydrogingerdiol (**11**) and (**12**) with hydrazine monohydrate yielded two detectable products in both cases. Regarding the derivatives of **12**, both products (**15** and **16**) were successfully isolated. On the other hand, in the case of compound **11**, only compound **14** was isolated in a reasonable amount. Reactions of **11** and **12** with hydroxylamine hydrochloride resulted in oxazines **17** and **18**, respectively. Unfortunately, some of the non-protonated aromatic carbons were not detected in the ^13^C NMR spectrum due to their long relaxation time after the irradiation, but the characteristic ^1^H proton resonances, HRMS spectra and spectral behaviour of the compounds allowed the establishment of their chemical structures.

**Scheme 3:**
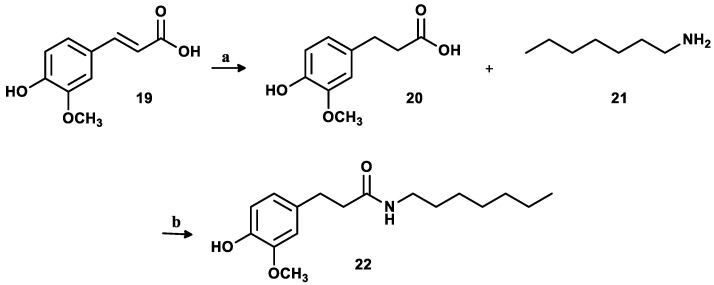
Preparation of the amide analogue of 6-paradol (**22**). Reaction conditions: **a**. H_2_/Pd/C/EtOAc/rt; **b**. DCC/DMAP/CH_2_Cl_2_/rt.

To investigate bioactivity changes upon replacing the keto group of compound **3** with an amide, we also prepared a capsaicin-like derivative (**22**). This was synthesised via a two-step procedure, starting with the catalytic hydrogenation of ferulic acid (**19**) to compound **20**, followed by its DCC/DMAP-mediated coupling with heptylamine (Scheme 3).

### 3.2. Predicted and Experimental Physicochemical and BBB Penetration-Related Characterisation

The characterisation of the 6-gingerol derivatives was started with the determination of their physicochemical and blood–brain barrier (BBB)-specific permeability properties, which are commonly used in early-stage drug discovery. The study was carried out on two levels: (a) an in silico approach, using lead optimisation parameters and the Central Nervous System Multiparameter Optimisation (CNS MPO) compliance introduced by Wager et al. [52]; and (b) experimentally, through determination of kinetic solubility and in vitro BBB permeability of the test compounds. Predicted and experimental data are shown in Table 1.

Combining Lipinski’s rule of five (Ro5) [53] and CNS MPO [52] criteria systems, the proton dissociation and lipophilicity properties of 6-gingerol derivatives were compared in a first approach. Regarding their acid–base character, the compounds can be classified into three groups: (i) monoprotic phenols (**1**–**6**, **17**, **18** and **22**), (ii) diprotic amphoterics (imidazole derivatives; **14**–**16**), and (iii) diprotic acids (gingerdione derivatives; **11**–**13**). The proton-dissociation behaviour of gingerdione derivatives is particularly interesting due to their tautomeric states shown in Scheme 3. The distribution % of the A-C tautomeric states of derivatives **11**–**13** was generated using Chemaxon Ltd.’s freely available Tautomer Generator plugin. This suggests compounds **11**–**13** to be mostly present in their enol forms (**A**:**B**, ~30:60%), while the dione forms with C-H acid function are much less expressed (**C**, ~10**%**). It is also important to note that, while in the case of the other gingerols (**1**–**6**, **14**–**22**) the strongest p*K_a,acid_* values refer to the aromatic OH function, the p*K_a,acid_* parameters indicate the proton-dissociation behaviour of the enol and dione C-H acid functions for **11**–**13**. Thus, based on these p*K_a,acid_* values, two conclusions can be made. First, in the case of **11**–**13**, the stronger acid function can be assigned to the enol and C-H acid moiety, and second, the acidic character of the **A**, **B** tautomeric forms is stronger than that of the **C** forms. In terms of lipophilicity, all 6-gingerol derivatives meet the drug-likeness criterion of Ro5 (log*P* < 5). At the same time, based on the two-level risk classification created by the lead-like [54] and CNS MPO criteria (log*D_pH7.4_*), **2**–**4** and **14**–**18** exceed (see Table 1, magenta: high violation), and **22** approach (yellow: moderate violation) the log*D_pH7.4_* violation limit. The lipophilicity values of the tautomers of **11**–**13** also show a marked difference. For all three compounds, the enol forms **A**/**B (11A**, **12A**, **13B)** are more lipophilic than the dione forms (**C**), which also manifests in the corresponding CNS MPO values. Regarding the next medchem parameter, the polar surface area (TPSA), all the tested gingerols satisfy both the Veber’s rule (30Å^2^ < TPSA < 140Å^2^) for bioavailability [55] and the CNS MPO range (40Å^2^ < TPSA < 90Å^2^) [52]. CNS MPO values (physchem-based CNS compliance) of gingerols were evaluated by a three-level classification system (green–yellow–magenta) for easier overview. Compounds **1**–**3**, **5**–**6**, **11**–**13**, **17** and **22** (green–yellow) can be considered as suitable candidates for further CNS-targeted preclinical studies. In the case of **11**–**13**, due to the lipophilicity and HBD differences of the tautomers, the **C** tautomeric forms carry the optimal CNS MPO character. In addition to the predicted parameters, experimental kinetic solubility (PBS, pH 7.4) and in vitro BBB-specific permeability (PAMPA-BBB) of the compounds were also determined. Also applying a three-level classification for the kinetic solubility values, the compounds **1**, **2**, **5**, **6**, **13**–**15** and **22** were in the acceptable range (greater than 100 μM). In parallel, due to limitations resulting from poor solubility, we could only determine the permeability of these gingerols using PAMPA-BBB study. In Table 1, compounds **1**, **2**, **5**, **6** and **22** are highlighted in green (P_e,BBB_ ≥ 25·10^−7^ cm/s), for which we identified increased BBB permeability. In addition to these compounds, **14**–**15** are also adequate for BBB penetration. In the case of **13**, the increased hydrophilic character may impair the BBB permeability.

### 3.3. Antiplatelet Aggregation and COX-1 Inhibition Activity of the Compounds

The arachidonic acid (AA)-cyclooxygenase-1 (COX-1) pathway plays an important role in platelet activation [56]. Aspirin, the standard antiplatelet drug, can prevent AA metabolism to thromboxane A2 by inhibiting COX-1, and thus exert antiplatelet effects [57]. To evaluate similar bioactivities of 6-gingerol (**1**) and its derivatives, the compounds were tested for their inhibitory effects on AA-induced platelet aggregation. Compounds **3**, **2**, **17**, **16**, and **13** showed the most promising results, with IC_50_ values of around 2–4 µM, respectively, while the effects of 6-gingerol (**1**) and aspirin were up to 22 and 50 times weaker, respectively (Table 2).

The antiplatelet mechanism of action of 6-gingerol and its derivatives has previously been suggested to be COX-1 inhibition [37,58,59]. The results obtained for the newly synthesised compounds are in accordance with this notion. The IC_50_ datasets of Table 2 give a linear correlation coefficient (R^2^) value of 0.887 (Supplementary Materials, Figure S26), strongly suggesting that COX-1 inhibition is indeed the mechanism behind the observed antiplatelet action.

Concerning structure–activity relationships, our results suggest the importance of an aromatic methoxy group, as seen by comparing the IC_50_ values of **11** vs. **12**, **14** vs. **15**, and **17** vs. **18**. Interestingly, this rule did not apply for compound **16**. This may underline a possible role of the Δ^1,2^ olefin in some cases, e.g., when it is conjugated with a pyrazole ring. Presence of the 5-OH group is highly unfavourable; its elimination (as in compounds **2**, **3**, and **4**) increased antiplatelet activity by ca. an order of magnitude, as well as its oxidation (compound **13** vs. **1**) or replacement by a heterocycle (e.g., compound **14** vs. **1**).

Compound **22**, in which the β-keto alcohol function of compound **1** was replaced by an amide group, showed only moderate activity. Notably, however, replacing the 3-oxo group by an oxime group led to only a slight, ca. 2-fold decrease in the antiplatelet activity (IC_50_ = 5.2 µM), while this chemical change opened the way to diverse further functionalisation possibilities.

Our results come in accordance with previous reports on compound **3**, i.e., 6-paradol that is naturally present in ginger roots.

Since the mid-2000s, the entropic or lipophilicity-driven lead optimisation constraint has mainly been observed in CNS-targeted drug discovery, significantly increasing the number of clinical candidates that were promiscuous or otherwise carrying off-target effects and toxicity risks [60]. To reduce this issue, several ligand efficiency metrics have been introduced [61], which can be used to filter out this effect. In this context, in our study, the IC_50_ data obtained on the two biological targets were evaluated using the ligand-lipophilic efficiency (LLE = pIC_50_−log*P*) metric [60]. After, a two-conditions-based lead selection was performed using the IC_50_ and LLE values, where the LLE values of 6-gingerol derivatives with an IC_50_ ≤ 10 µM and an LLE value higher than 1.0 were highlighted (Table 2, compounds coloured green for LLE_Antipatelet_: **2**, **3**, **11**, **13** and **17** and LLE_COX-1_: **3**). Summarizing the data in Table 1 and Table 2, due to the strict selection, only compound **2** can be assigned as a primary candidate for further preclinical studies, while compounds **3**, **11** and **17** are potential leads that require an appropriate formulation to improve their aqueous solubility. From the point of view of lead optimisation, compound **3** is particularly interesting, satisfying the LLE/IC_50_ screening criteria for both biological targets. Compound **13** can also be identified as a secondary lead, for which the further goal may be fine-tuning the BBB permeability property.

### 3.4. Molecular Docking

Compounds were docked using AutoDock4 into the human COX-1 enzyme crystal structure retrieved from the Protein Data Bank (PDB ID: 6Y3C). Grid parameters were set to centre at residue Ser530 and to include residues Tyr385, Arg120, and Tyr348 that are in the COX-1 binding pocket [62]. In the cases of compounds **11**, **12**, and **13**, the tautomeric forms **A–C** were subjected to in silico docking. Detailed results of the docking study are provided as Supplementary Materials, Table S1.

**Figure 1:**
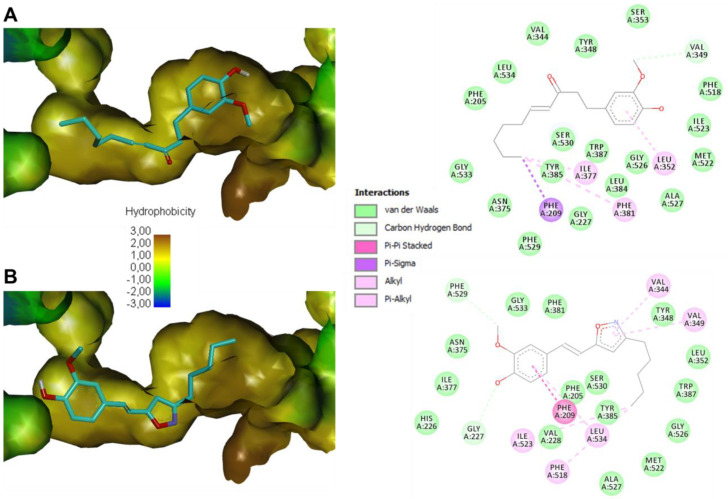
The binding mode of compounds **2** (**A**) and **17** (**B**) on *h*-COX-1 enzyme (PDB ID: 6Y3C) visualised with Discovery studio visualizer (21.1.0.20298); 3D orientation with the enzyme’s hydrophobicity surface to the left and 2D representation of the observed interactions to the right. Colour codes for **A** and **B** are identical.

The highest binding affinity (−9.5 Kcal/mol) was found for the new isoxazole-containing compound **17**, which was also among the most potent antiplatelet derivatives. L-Toth et al. discussed the importance of Tyr385 and Ser530 in the irreversible binding of aspirin to COX-1 active site; notably, the docking results showed hydrogen bonding interactions with Ser530 in the case of compound **1**, **4**, and **6**, while compounds **11B**, **12A**, and **22** appeared to interact via hydrogen bonds with Tyr385 (Table S1). Interestingly, compound **17** did not show any of the above-mentioned interaction with either of these amino acids (Figure 1).

### 3.5. Antioxidant Assay

Antioxidant activity was assessed using multiple models, including DDPH, ORAC, ONOO^−^ scavenging, and XO inhibition assays; results are shown in Table 3. Compounds **5**, **17**, **4**, **1**, and **11** showed the best activity in the diphenyl-2-picrylhydrazyl (DPPH) scavenging capacity assay. Among these, compound **5** is clearly the most promising antioxidant lead, considering its DPPH-scavenging IC_50_ value, its ORAC value, which is more than twice as potent as that of Trolox, and its predicted and experimental pharmacokinetic parameters. Interestingly, compound **6**, the three-epimer variant of **5**, showed only ca. half of the activity of **5** in the DPPH assay and was also weaker in terms of ORAC. Similar to the results obtained for the antiplatelet and COX-1 inhibition assay, the importance of an aromatic methoxy group for potent DPPH scavenging activity was highlighted; except for compound **16**, all compounds without this moiety (**12**, **15**, and **18**) were inactive in this regard. Concerning their ORAC values, however, compounds **12** and **16** were the most potent among all compounds, which highlights the complementary value of these two bioassays to evaluate free radical scavenging activity of small molecule antioxidants.

## 4. Conclusions

In this study, a combination semi and total synthetic strategy was adopted to prepare fourteen 6-gingerol derivatives including eight new compounds, which were subsequently characterised as antiplatelet and COX-1 inhibitor agents, and free radical scavenger or XO inhibitor antioxidants. The compounds’ pharmacodynamic and pharmacokinetic characterisation revealed 6-shogaol (**2**) to be the best lead as a cardiovascular protective agent, and compounds **3**, **11**, and **17** as new starting points for hit-to-lead optimisation. The 3,5-diol compound **5** was identified as a more potent and less promiscuous antioxidant than its parent compound 6-gingerol (**1**) or its three-epimer compound **6**. Due to its favourable pharmacokinetic parameters, compound **5** is also suggested as a potential CNS-specific antioxidant. Further studies to evaluate this notion are to be conducted soon.

## Figures and Tables

**Table 1:** In silico and experimental data for physicochemical and BBB permeability characterisation. Results are given as mean ± standard error of the mean (SEM); *n* = 3.

		Predicted Values ^a^					Experimental Data	
Cmpds ID	Tautomer Distribution (%) ^b^	p*K_a,base_*/p*K_a,acid_^c^*	log*P*/log*D_pH7.4_*	TPSA	HBD/HBA	CNS MPO ^d^ [52]	Kinetic Solubility ^e^ (μM)	PAMPA-BBB ^e’^ P_e_ (·10^−7^ cm/s)/MR (%)
**1**		-/10.0	2.9/2.9	66.8	2/4	5.06	>500	35.2 ± 2.4/23.8 ± 1.5
**2**		-/10.0	4.2/4.2	46.5	1/3	4.18	110.2 ± 3.8	31.7 ± 3.6/20.9 ± 3.9
**3**		-/10.0	4.1/4.1	46.5	1/3	4.19	45.2 ± 2.7	-/11.0 ± 0.7
**4**		-/10.1	4.8/4.8	62.1	2/4	3.60	75.1 ± 7.3	-/4.0 ± 11.1
**5**		-/10.1	3.0/3.0	69.9	3/4	4.72	>500	33.9 ± 2,5/9.1 ± 7.6
**6**		-/10.1	3.0/3.0	69.9	3/4	4.72	460.7 ± 10.3	27.4 ± 1.6/18.5 ± 2.7
**11A**	31	-/8.3	3.5/3.5	66.8	2/4	4.81	54.0 ± 1.3	-/-
**11B**	60	-/8.3	3.2/3.2	66.8	2/4	4.53		
**11C**	9	-/8.7	3.3/3.3	63.6	1/4	5.01		
**12A**	38	-/8.3	3.9/3.8	57.5	2/3	4.62	15.6 ± 0.1	-/-
**12B**	54	-/8.4	3.4/3.4	57.5	2/3	4.15		
**12C**	8	-/8.9	3.5/3.5	54.4	1/3	4.75		
**13A**	31	-/8.7	3.4/3.4	66.8	2/4	4.58	314.6 ± 23.7	-/-
**13B**	60	-/8.7	3.5/3.4	66.8	2/4	4.56		
**13C**	9	-/9.4	3.3/3.3	63.6	1/4	4.98		
**14**		3.8/10.1	4.7/4.7	58.1	2/4	3.67	276.7 ± 5.5	15.8 ± 0.8/33.6 ± 4.6
**15**		3.9/10.1	4.9/4.9	48.9	2/3	3.57	202.9 ± 5.5	20.7 ± 4.3/3.1 ± 5.7
**16**		3.1/10.0	4.8/4.8	48.9	2/3	3.59	89.4 ± 3.1	-/-
**17**		-/9.9	4.4/4.4	55.5	1/4	4.04	10.9 ± 2.0	-/-
**18**		-/9.8	4.8/4.8	46.3	1/3	3.87	<LOD	-/-
**22**		-/10.1	3.6/3.6	58.6	2/4	4.36	482.6 ± 18.7	32.2 ± 3.4/12.1 ± 5.2
Aspirin		-/3.5	1.4/-1.7	63.6	1/4	5.75	-	-

^a^ Predicted values calculated by ACD/Labs Percepta software [46], ^b^ Generated by Chemaxon Tautomer Generator [47], ^c^ strongest acidic p*K_a_*, ^d^ CNS MPO were determined using predicted p*K_a,basic_*, log*P*/log*D_pH7.4_* (classic and consensus settings, respectively), TPSA and HBD values, ^e/e’^ after 2 h/4 h, at 37 °C in PBS, pH 7.4. *Colours of classification systems*: log*D_pH7.4_*: moderate violation (yellow) ≥ 3.5, high violation (magenta) ≥ 4.0; CNS MPO: low (magenta) ≤ 4.0, moderate (yellow) ≤ 4.7, good (green) > 4.7; Kin.Sol.: low (magenta) ≤ 100, moderate (yellow) ≤ 300, good (green) > 300; Increased BBB-permeability (green) > 25.

**Table 2:** Antiplatelet and Cyclooxygenase-1 (COX-1) inhibition assay results. For antiplatelet activity, washed human platelets were treated with gingerol derivatives for 3 min and then stimulated with arachidonic acid (100 μM); data are presented as mean ± SEM, *n* = 3 for antiplatelet and *n* = 2 for COX-1 inhibition assay; aspirin was used as a positive control.

Compound	Antiplatelet IC_50_ (µM)	$LLE_{\left(Antiplatelet\right)}^{a}$	COX-1 IC_50_ (µM)	$LLE_{\left(COX-1\right)}^{a}$
**1**	45.9 ± 5.1	1.46	62.5 ± 23.8	1.30
**2**	2.8 ± 0.5	1.40	9.8 ± 0.6	0.81
**3**	2.1 ± 1.0	1.56	4.4 ± 0.2	1.26
**4**	5.2 ± 0.4	0.47	5.2 ± 0.3	0.48
**5**	51.7 ± 2.7	1.26	54.3 ± 6.5	1.27
**6**	45.1 ± 6.0	1.32	76.2 ± 0.3	1.12
**11(A)** ^b^	4.1 ± 1.0	1.89 ^b^	23.1 ± 9.3	1.14
**12(A)** ^b^	71.7 ± 28.3	0.24 ^b^	>200	-
**13(B)** ^b^	3.6 ± 0.9	1.94 ^b^	11.8 ± 5.4	1.53
**14**	4.1 ± 1.2	0.72	3.6 ± 0.2	0.74
**15**	>100	-	>200	-
**16**	3.5 ± 0.9	0.63	17.5 ± 0.1	−0.04
**17**	3.1 ± 0.9	1.08	5.85 ± 0.04	0.83
**18**	32.0 ± 10.1	−0.27	>200	-
**22**	35.9 ± 23.7	0.80	>100	-
**Aspirin**	106.0 ± 20.2	2.58	-	-

^a^ Ligand–lipophilic efficiency: LLE = pIC_50_—log*P*_predicted_. For LLE, green colouring indicates a satisfactory level (LLE_(Antiplatelet)_ ≥ 1.5 or LLE_(COX-1)_ ≥ 1.0 and IC_50_ ≤ 10 µM). ^b^ For the sake of strict characterisation, the tautomer with the highest log*P* value (see Table 1) was included in the LLE calculation for compounds **11**–**13**, i.e., the value for the worst possible case is shown in the table.

**Table 3:** The antioxidant activity results of 6-gingerol and its derivatives. Values are given as mean ± standard error of the mean (SEM). ORAC assay results are given in Trolox equivalents (TE), ONOO^−^ scavenging and xanthine oxidase (XO) inhibition assay results are given in % inhibition at concentrations of 500 and 100 µM, respectively; *n* = 2 for DPPH; *n* = 3 for ORAC, ONOO^−^ and XO.

	DDPH		ORAC TE	ONOO^–^ Scavenging (%)	XO Inhibition (%)
	IC_50_ (µM)	LLE ^a^			
**1**	8.92 ± 0.46	2.15	1.30 ± 0.04	<5.0	<5.0
**2**	11.41 ± 0.49	0.75	1.10 ± 0.03	<5.0	<5.0
**3**	9.43 ± 0.16	0.93	1.36 ± 0.02	<5.0	<5.0
**4**	8.56 ± 0.07	0.27	0.47 ± 0.09	<5.0	10.56 ± 2.30
**5**	6.51 ± 0.28	2.19	2.30 ± 0.05	<5.0	<5.0
**6**	13.82 ± 0.03	1.86	1.12 ± 0.03	<5.0	<5.0
**11**	9.04 ± 0.19	1.54	1.98 ± 0.12	<5.0	<5.0
**12**	>200	-	2.89 ± 0.49	<5.0	<5.0
**13**	10.86 ± 0.69	1.46	2.60 ± 0.06	<5.0	<5.0
**14**	16.16 ± 0.46	0.09	0.44 ± 0.07	<5.0	10.13 ± 1.65
**15**	>200	-	1.00 ± 0.02	<5.0	12.30 ± 0.90
**16**	18.98 ± 1.63	−0.08	2.88 ± 0.16	<5.0	16.01 ± 4.05
**17**	8.13 ± 0.21	0.69	0.77 ± 0.20	38.40 ± 3.05	<5.0
**18**	>100	-	1.76 ± 0.06	5.64 ± 1.31	<5.0
**22**	14.07 ± 0.21	1.25	1.62 ± 0.004	<5.0	<5.0
**allopurinol**	-	-	-	-	98.80 ± 0.11

^a^ Ligand–lipophilic efficiency: LLE = pIC_50_—log*P*_predicted_. For LLE, green colouring indicates a satisfactory level (LLE ≥ 1.5 and IC_50_ ≤ 10 µM).

## Data Availability

The data presented in this study are available in the article and Supplementary Materials.

## Supplementary Materials

The following Supplementary Materials can be downloaded at: https://www.mdpi.com/article/10.3390/antiox12030744/s1, Figures S1–S8: HRMS spectra for compounds **4**, **12**, **14**–**18** and **22**, Figures S9–S24: ^1^H and ^13^C NMR spectra for compounds **4**, **12**, **14**–**18** and **22**, Figure S25: Correlation between the antiplatelet and COX-1 inhibitory activities of the tested compounds; Table S1: Docking results obtained using AutoDockTools 1.5.7 and Discovery Studio visualizer (21.1.0.20298).
