# Supplementary material for: Preparation and Evaluation of 6-Gingerol Derivatives as Novel Antioxidants and Antiplatelet Agents
Source: Antioxidants (Basel). 2023 Mar 17;12(3):744. doi: 10.3390/antiox12030744 (PMC10045534; doi:10.3390/antiox12030744)
Supplement: Supplementary file 1 [file antioxidants-12-00744-s001.zip › antioxidants-2256440-supplementary.pdf]

Supplementary Materials to

Preparation and Evaluation of 6-Gingerol Derivatives as Novel Antioxidants and Antiplatelet Agents

Sara H. H. Ahmed [1,†,‡], Tímea Gonda [1,†], Orinamhe G. Agbadua [1], Gábor Girst [1], Róbert Berkecz [2], Norbert Kúsz [1], Meng-Chun Tsai [3], Chin-Chung Wu [3], György T. Balogh [4,5] and Attila Hunyadi [1,6,*]

1 Institute of Pharmacognosy, University of Szeged, H-6720 Szeged, Hungary
2 Institute of Pharmaceutical Analysis, University of Szeged, H-6720 Szeged, Hungary
3 Graduate Institute of Natural Products, Kaohsiung Medical University, Kaohsiung 807, Taiwan
4 Institute of Pharmacodynamics and Biopharmacy, University of Szeged, H-6720 Szeged, Hungary
5 Department of Chemical and Environmental Process Engineering, Budapest University of Technology and Economics, H-1111 Budapest, Hungary
6 Interdisciplinary Centre of Natural Products, University of Szeged, H-6720 Szeged, Hungary
* Correspondence: hunyadi.attila@szte.hu; Tel.: +36-62545557
† These authors contributed equally to this work.
‡ On leave from the Department of Pharmaceutical Chemistry, Faculty of Pharmacy, University of Khartoum, 11111 Khartoum, Sudan.

Table of contents

Figure S1. HRMS spectrum of compound **4**
Figure S2. HRMS spectrum of compound **12**
Figure S3. HRMS spectrum of compound **14**
Figure S4. HRMS spectrum of compound **15**
Figure S5. HRMS spectrum of compound **16**
Figure S6. HRMS spectrum of compound **17**
Figure S7. HRMS spectrum of compound **18**
Figure S8. HRMS spectrum of compound **22**
Figure S9. ^{1}H-NMR spectrum of compound **4**
Figure S10. ^{13}C-NMR spectrum of compound **4**

Figure S11. ^{1}H-NMR spectrum of compound **12**
Figure S12. ^{13}C-NMR spectrum of compound **12**
Figure S13. ^{1}H-NMR spectrum of compound **14**
Figure S14. ^{13}C-NMR spectrum of compound **14**
Figure S15. ^{1}H-NMR spectrum of compound **15**
Figure S16. ^{13}C-NMR spectrum of compound **15**
Figure S17. ^{1}H-NMR spectrum of compound **16**
Figure S18. ^{13}C-NMR spectrum of compound **16**
Figure S19. ^{1}H-NMR spectrum of compound **17**
Figure S20. ^{13}C-NMR spectrum of compound **17**
Figure S21. ^{1}H-NMR spectrum of compound **18**
Figure S22. ^{13}C-NMR spectrum of compound **18**
Figure S23. ^{1}H-NMR spectrum of compound **22**
Figure S24. ^{13}C-NMR spectrum of compound **22**
Figure S25. Correlation between the antiplatelet and COX-1 inhibitory activities of the tested compounds
Table 1. Docking results obtained using AutoDockTools 1.5.7 and Discovery Studio visualizer (21.1.0.20298)

Figure S1. HRMS spectrum of compound **4**

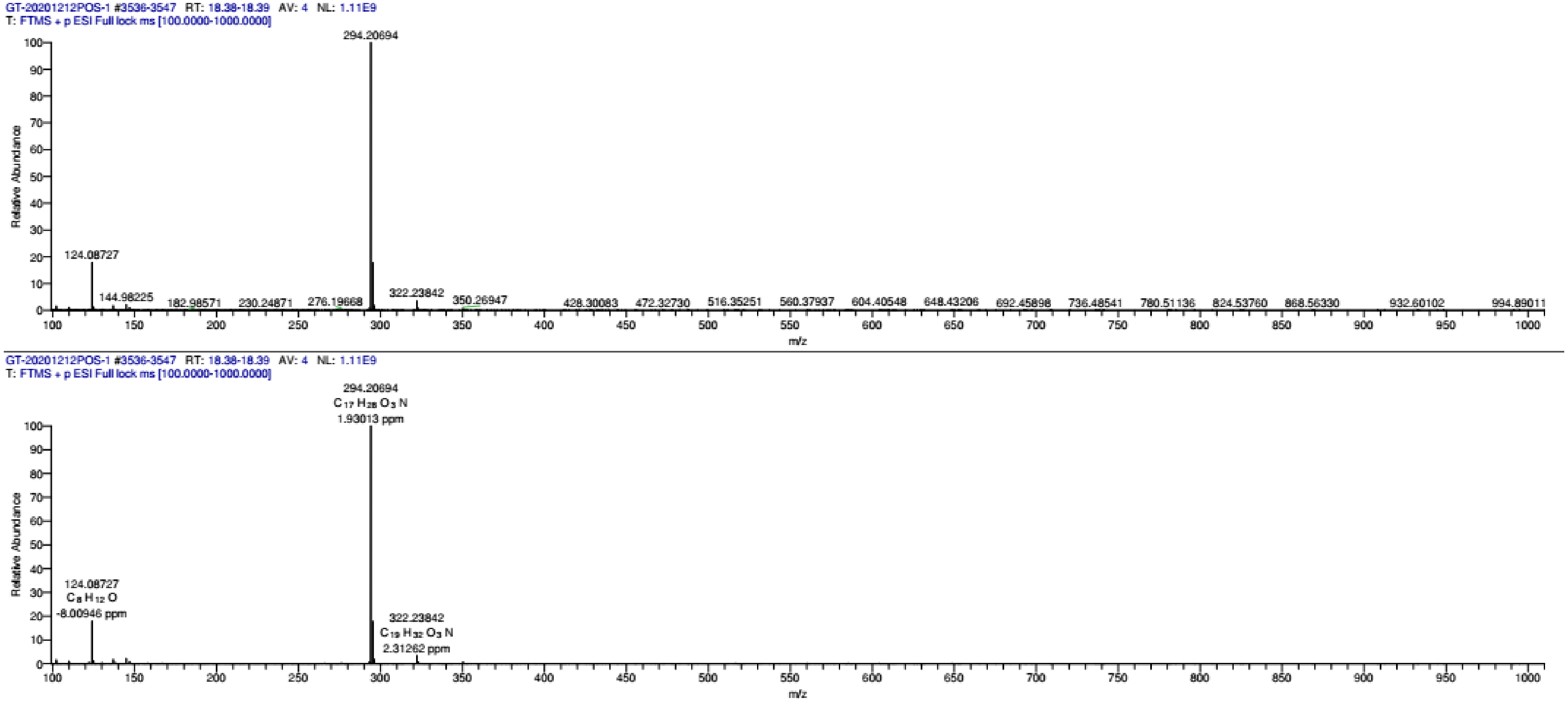

Figure S2. HRMS spectrum of compound **12**

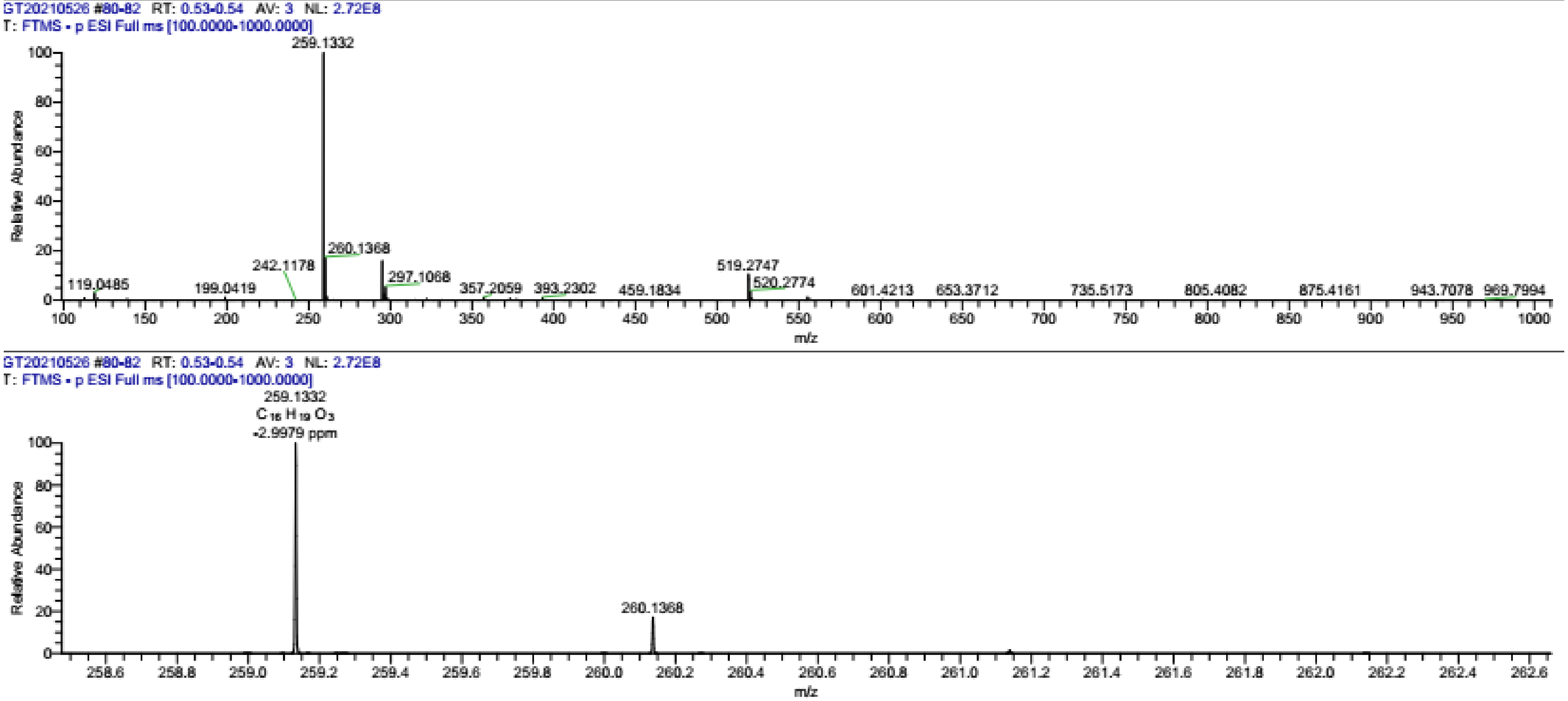

Figure S3. HRMS spectrum of compound **14**

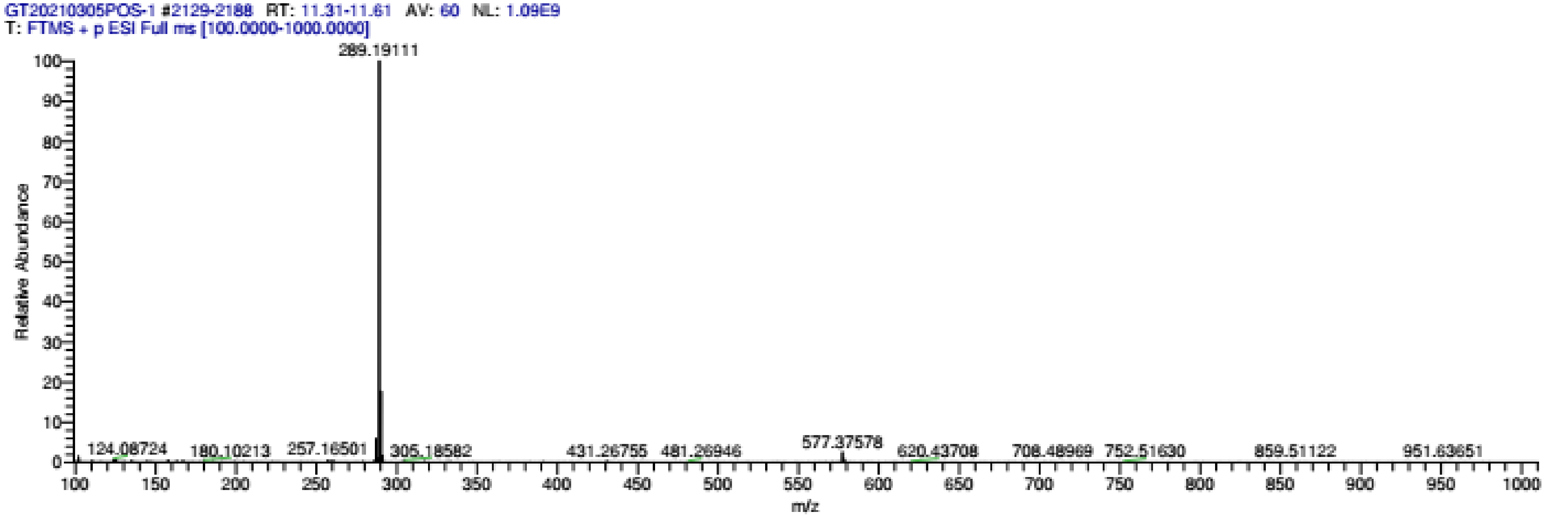

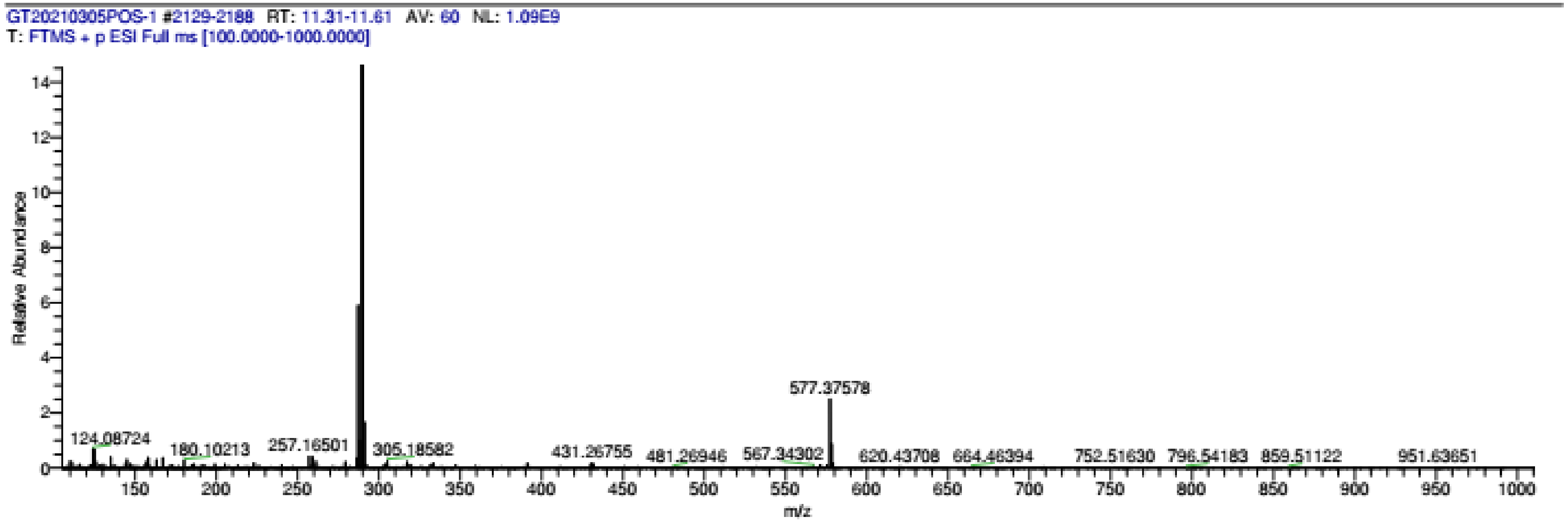

Figure S4. HRMS spectrum of compound **15**

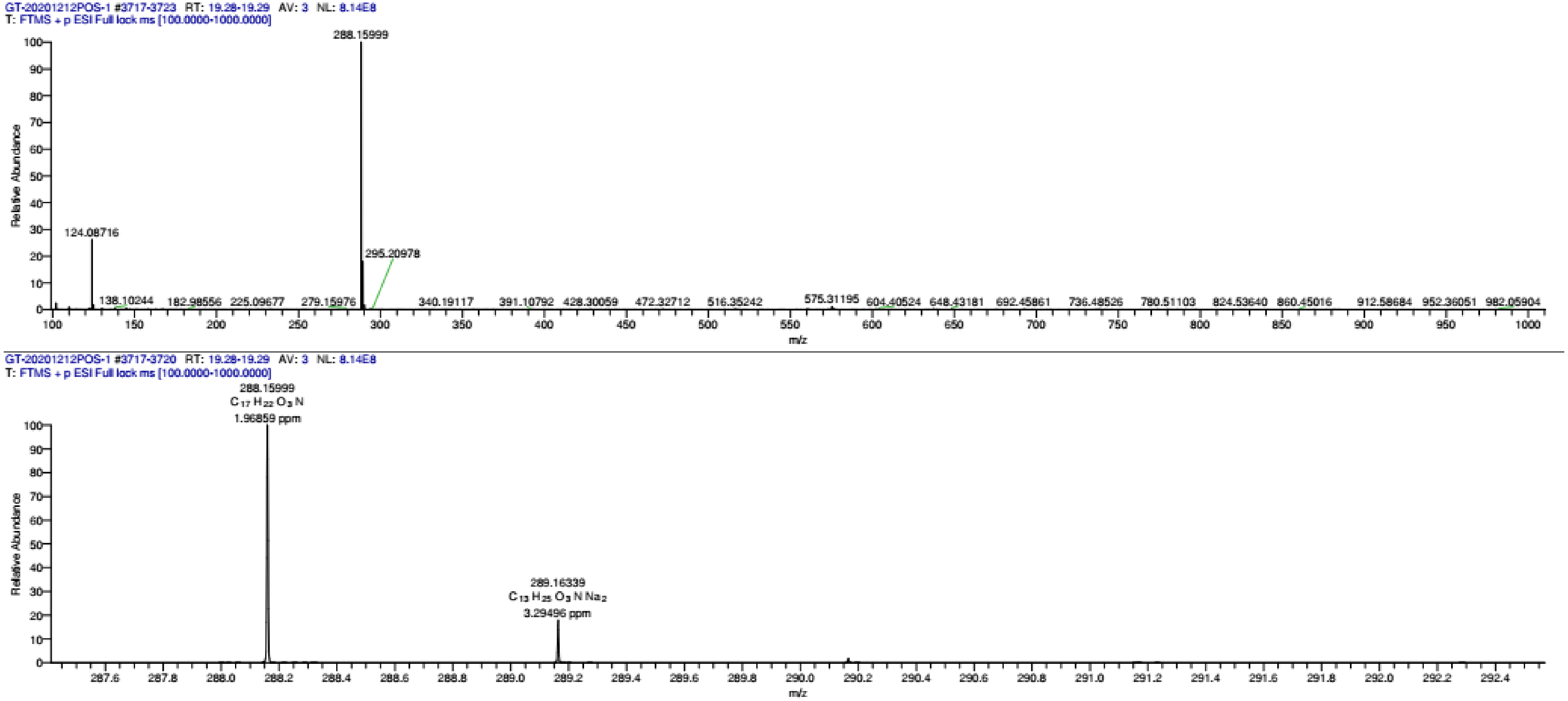

Figure S5. HRMS spectrum of compound **16**

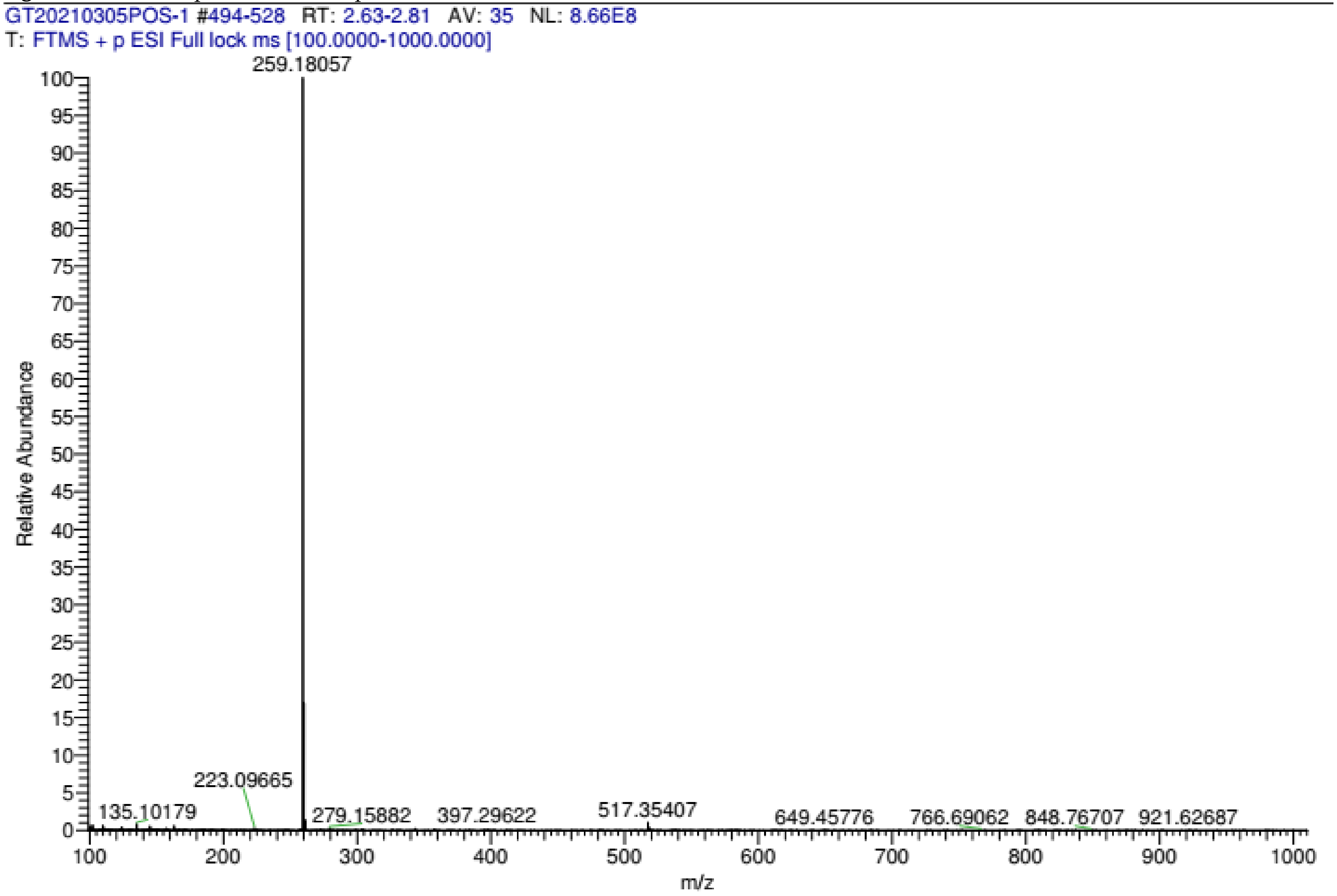

Figure S6. HRMS spectrum of compound **17**

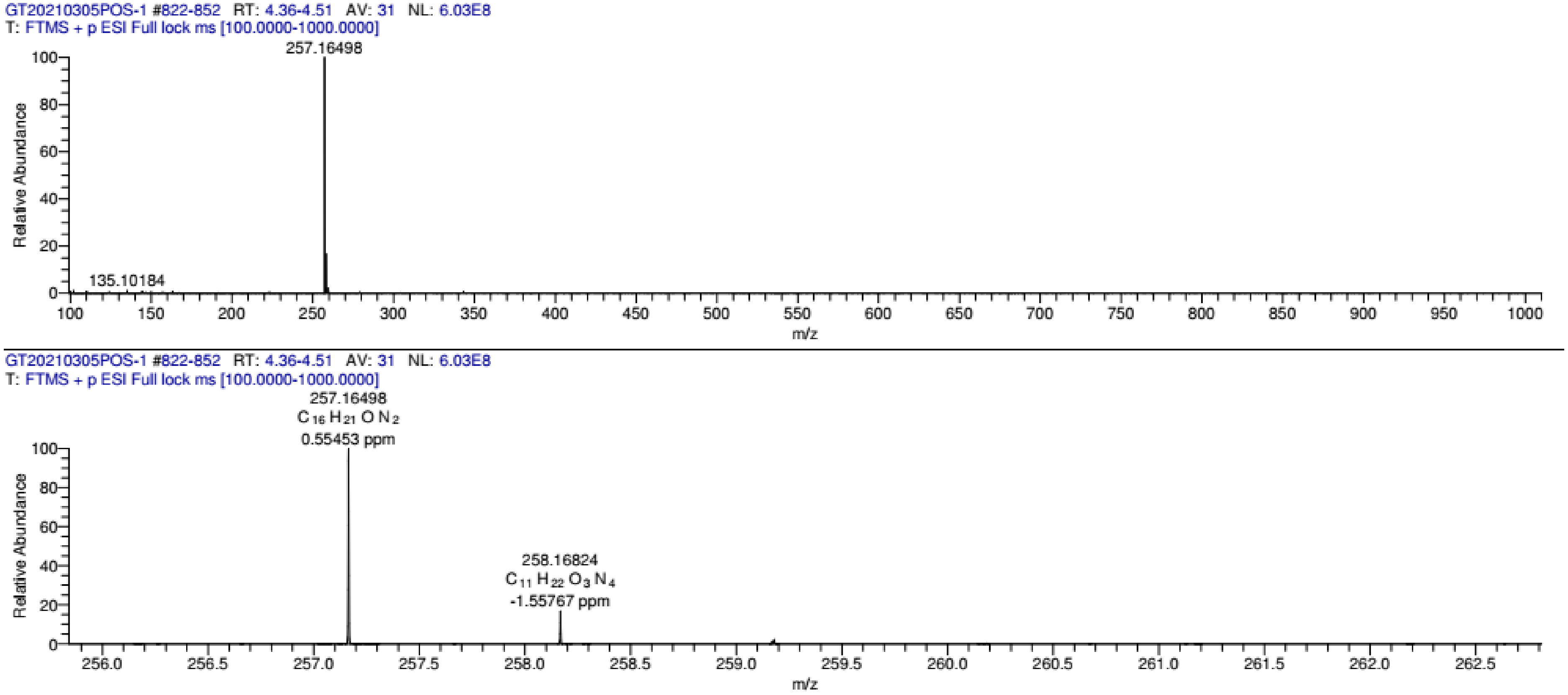

Figure S7. HRMS spectrum of compound **18**

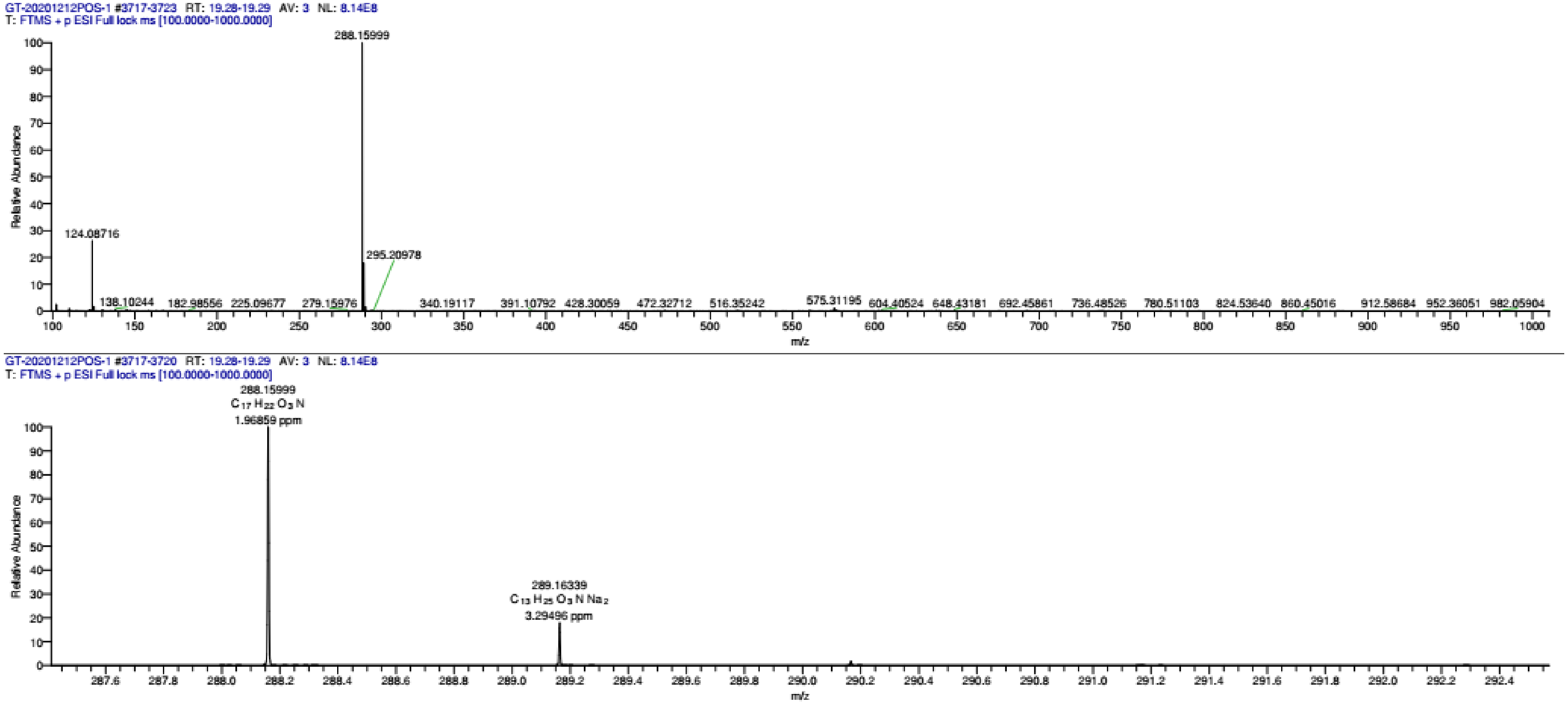

Figure S8. HRMS spectrum of compound **22**

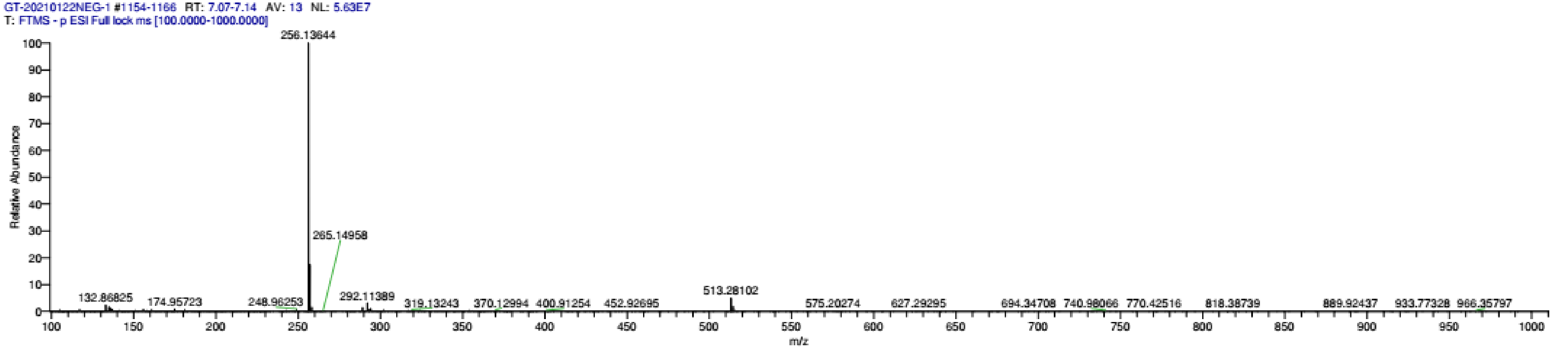

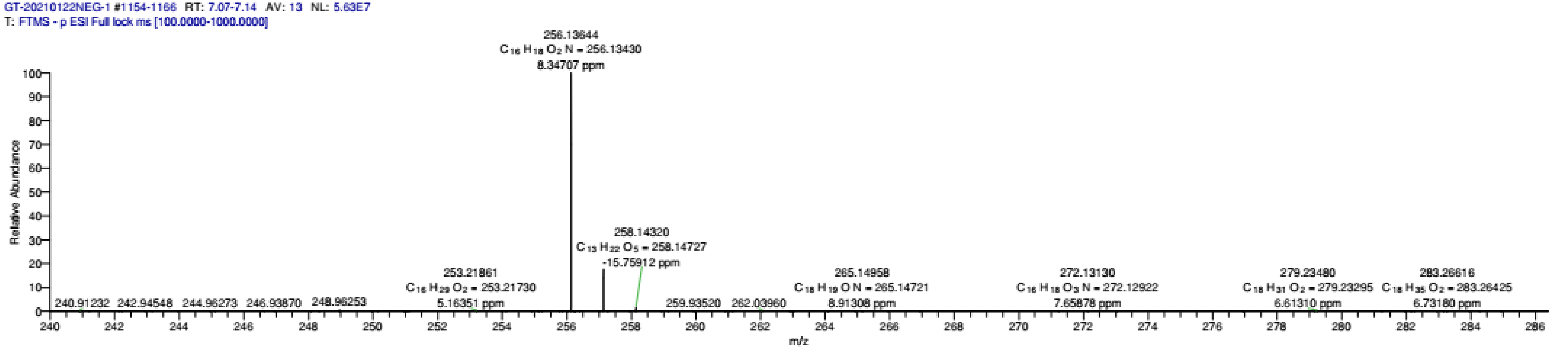

Figure S9. ^{1}H-NMR spectrum of compound **4**

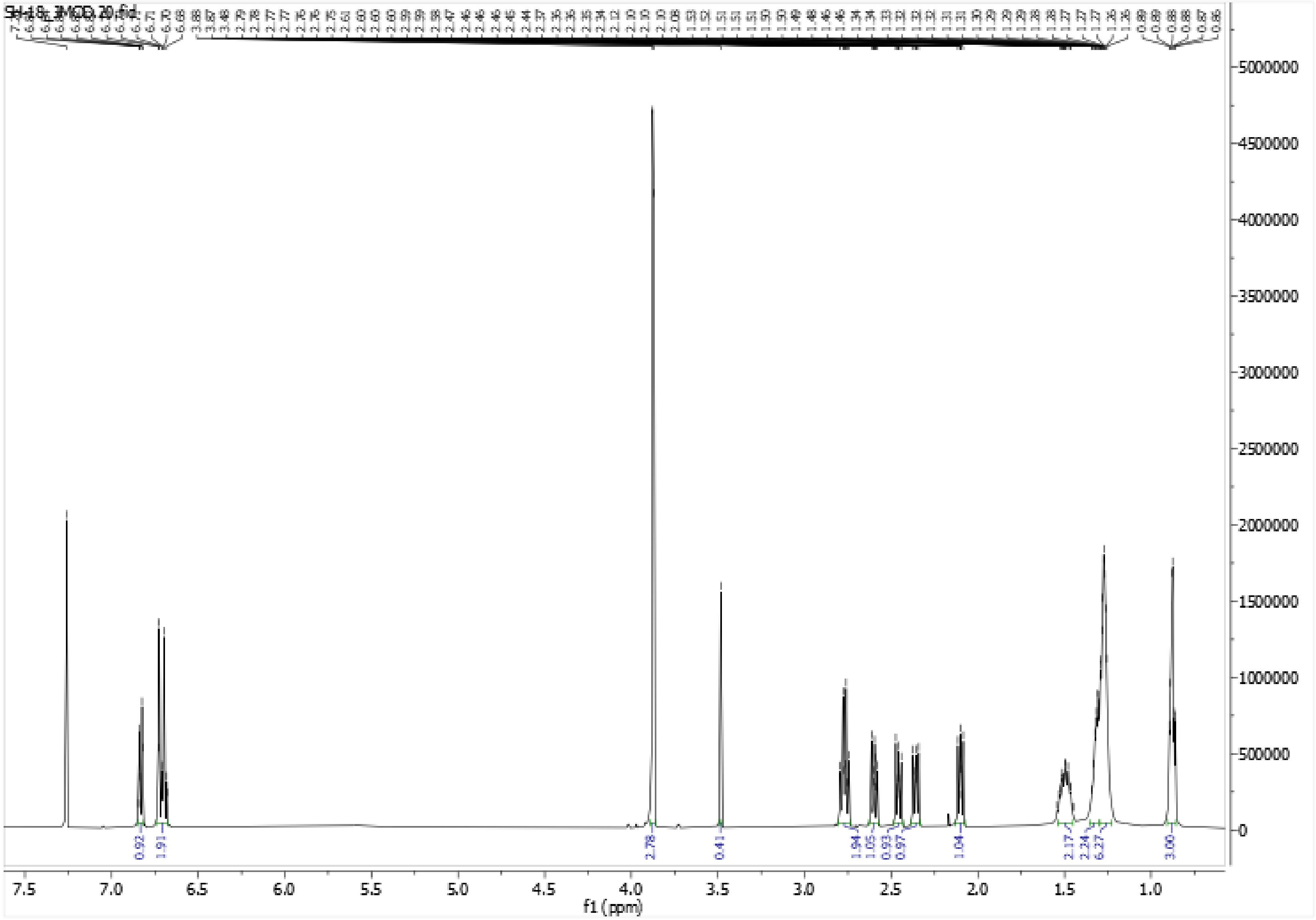

Figure S10. ^{13}C-NMR spectrum of compound **4**

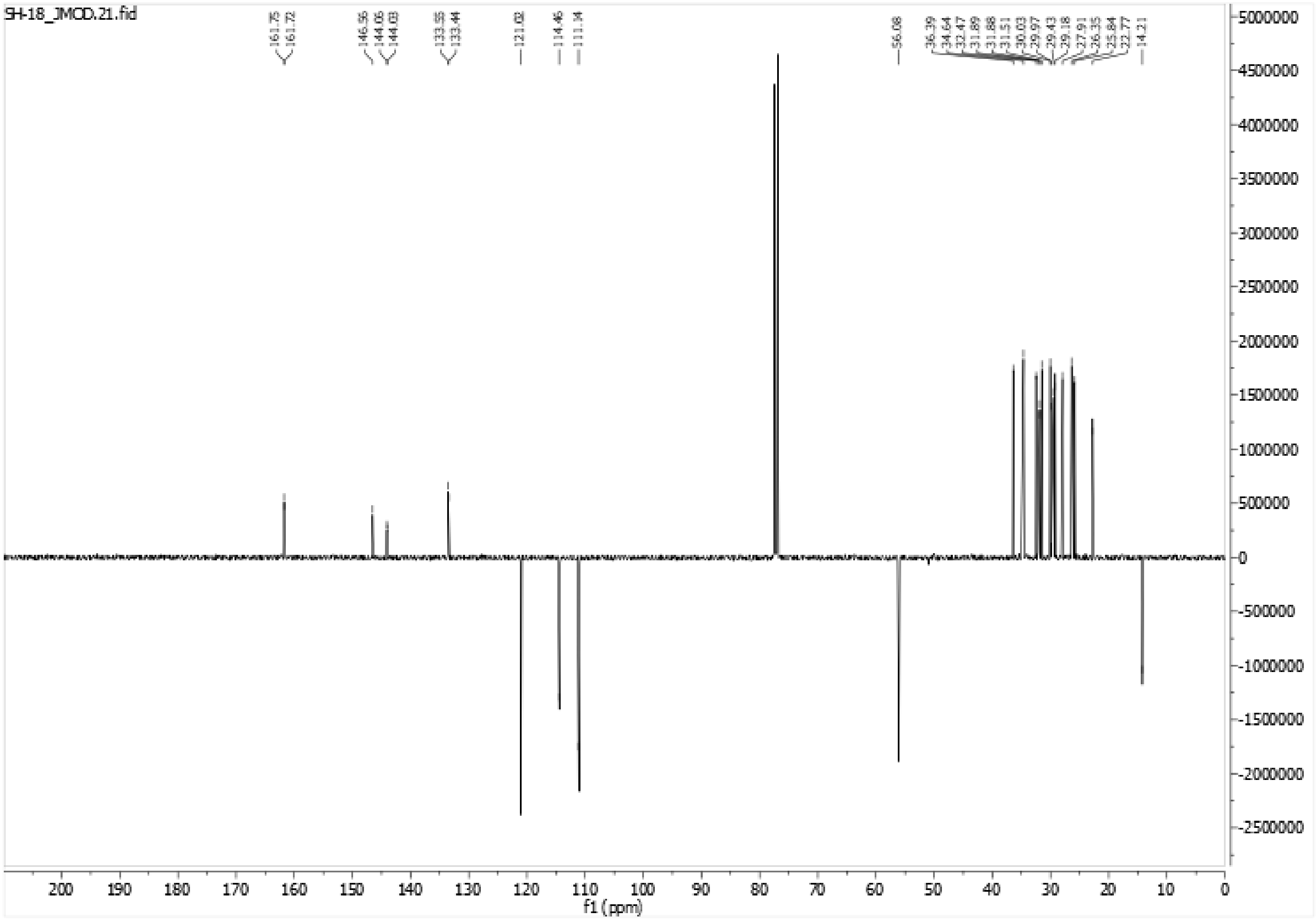

Figure S11. ^{1}H-NMR spectrum of compound **12**

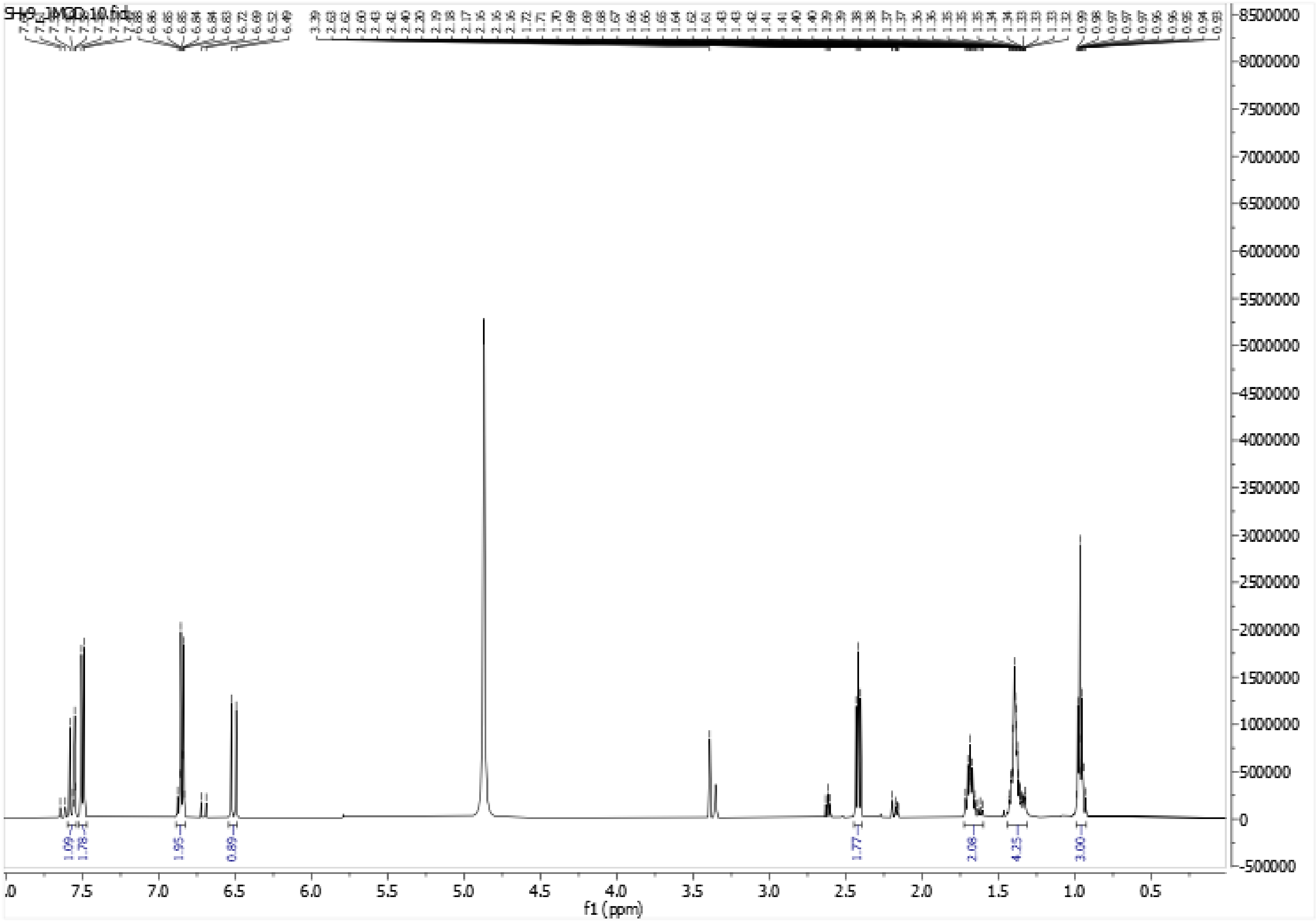

Figure S12. ^{13}C-NMR spectrum of compound **12**

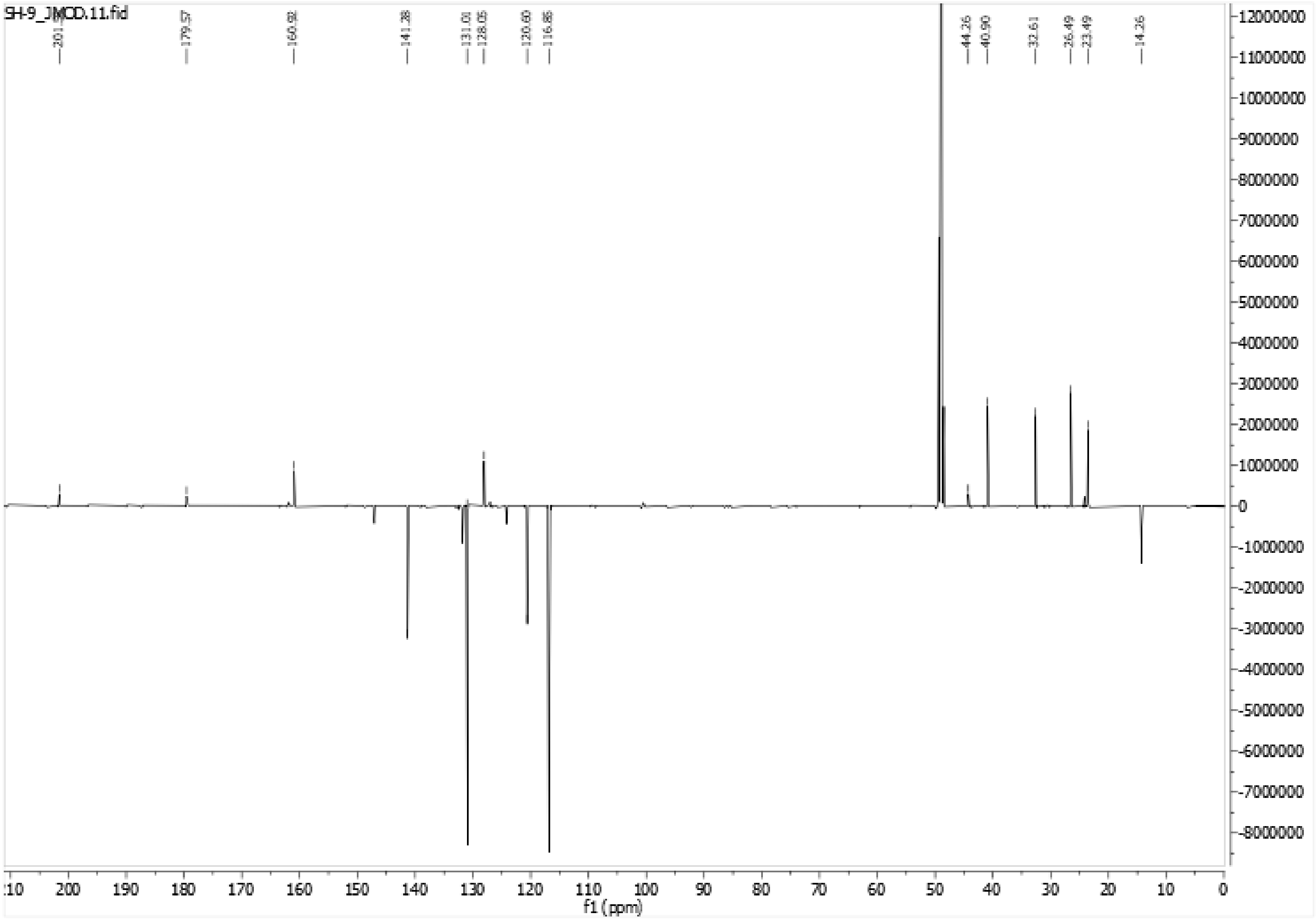

Figure S13. ^{1}H-NMR spectrum of compound **14**

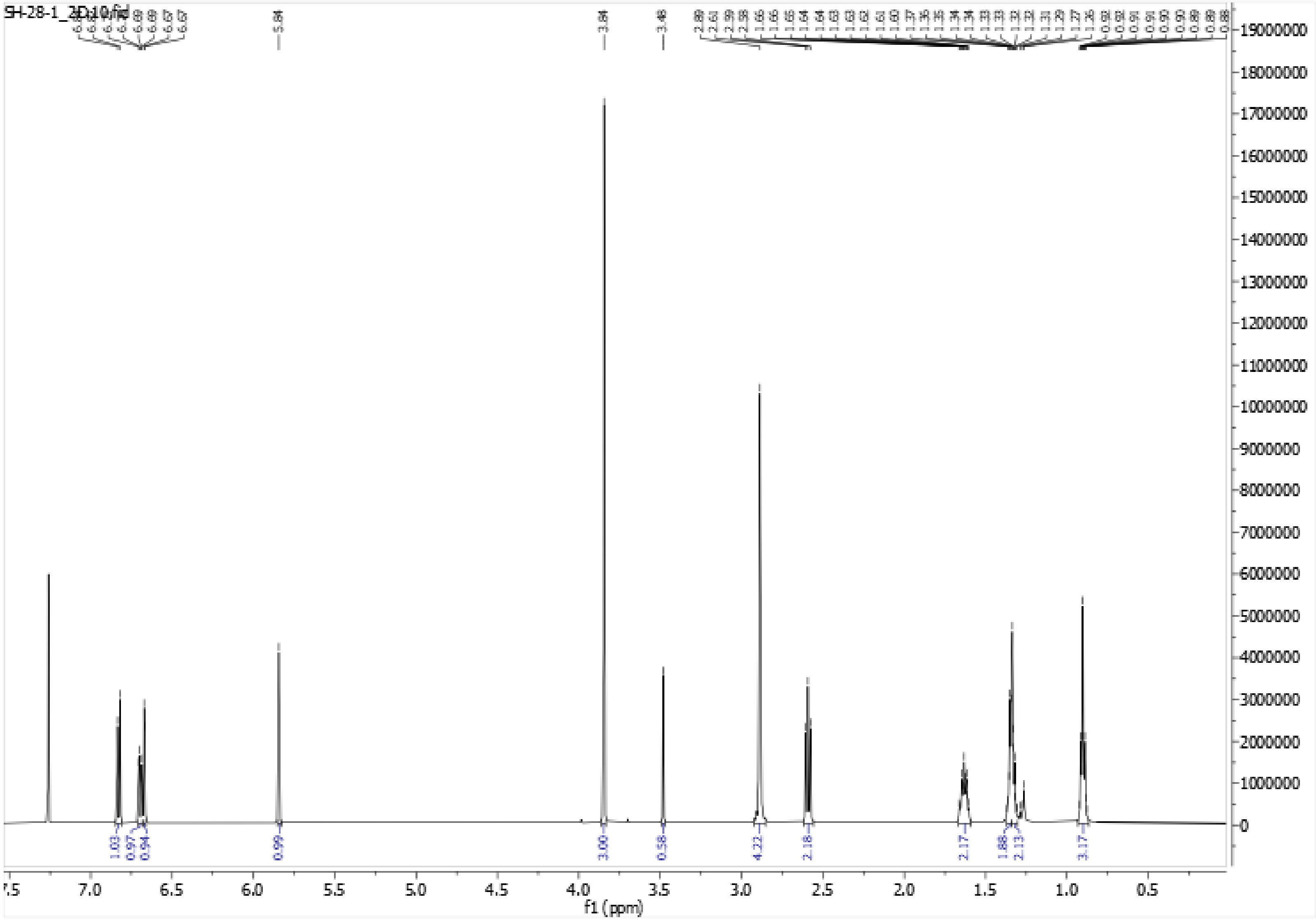

Figure S14. ^{13}C-NMR spectrum of compound **14**

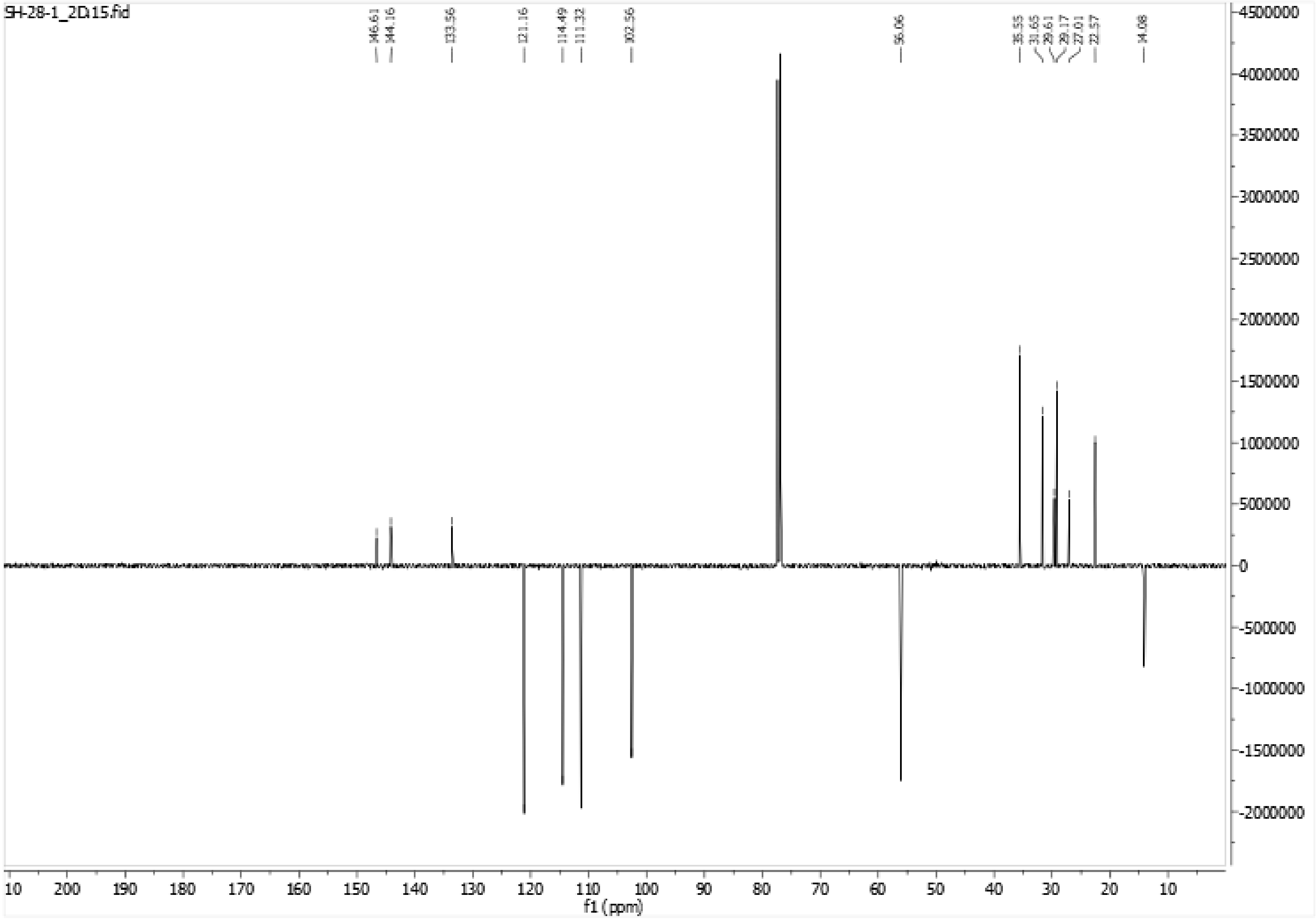

Figure S15. ^{1}H-NMR spectrum of compound **15**

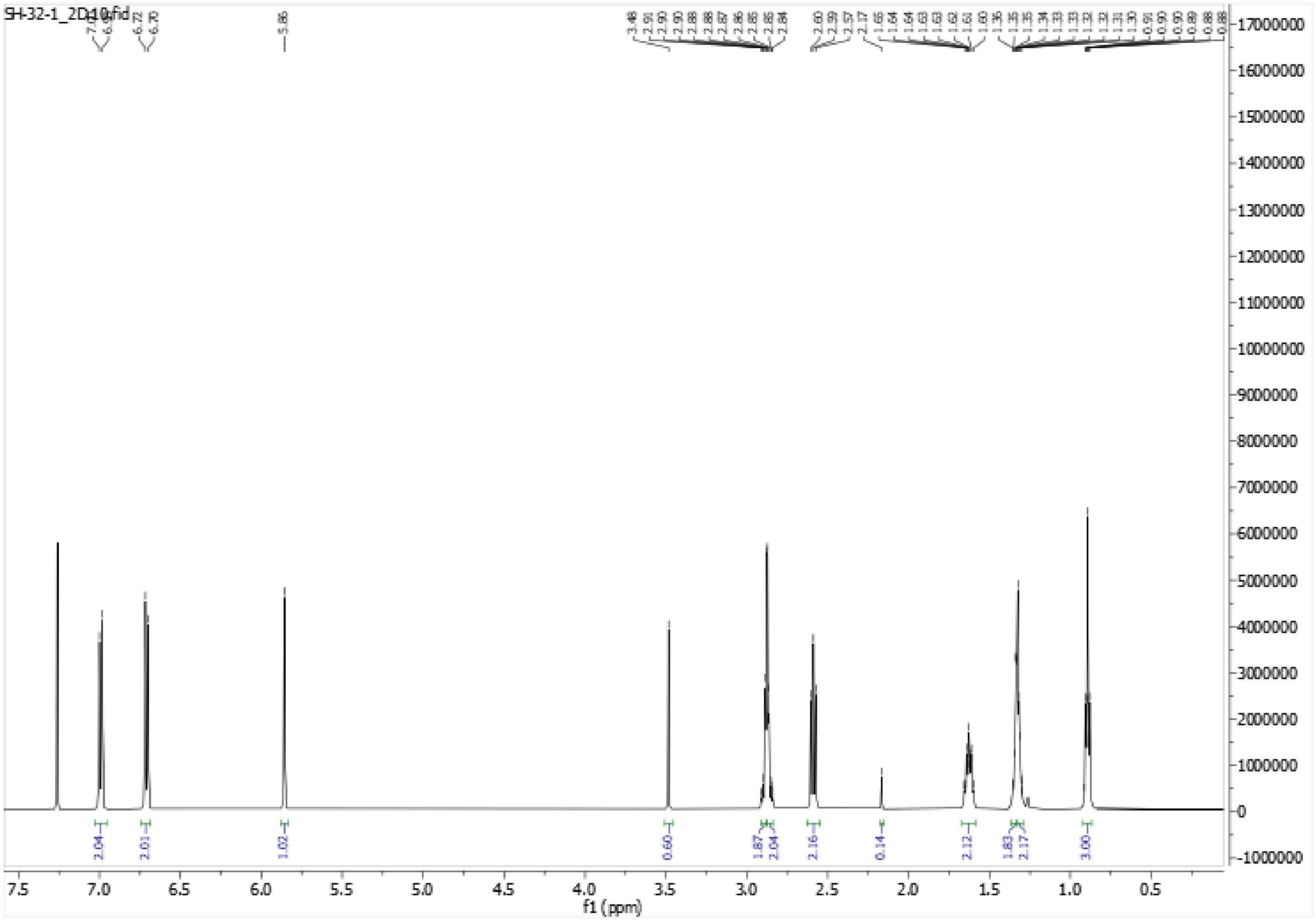

Figure S16. ^{13}C-NMR spectrum of compound **15**

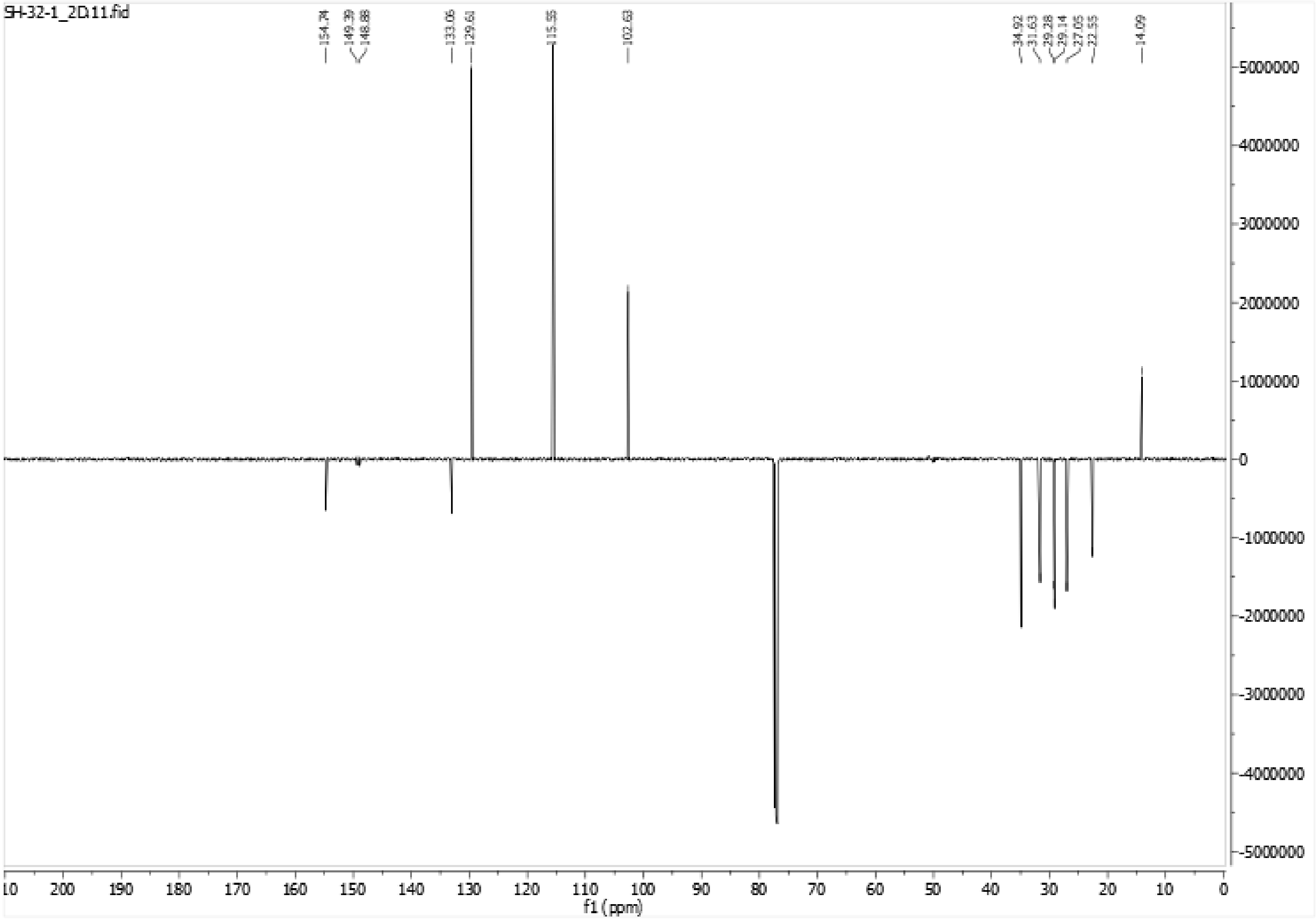

Figure S17. ^{1}H-NMR spectrum of compound **16**

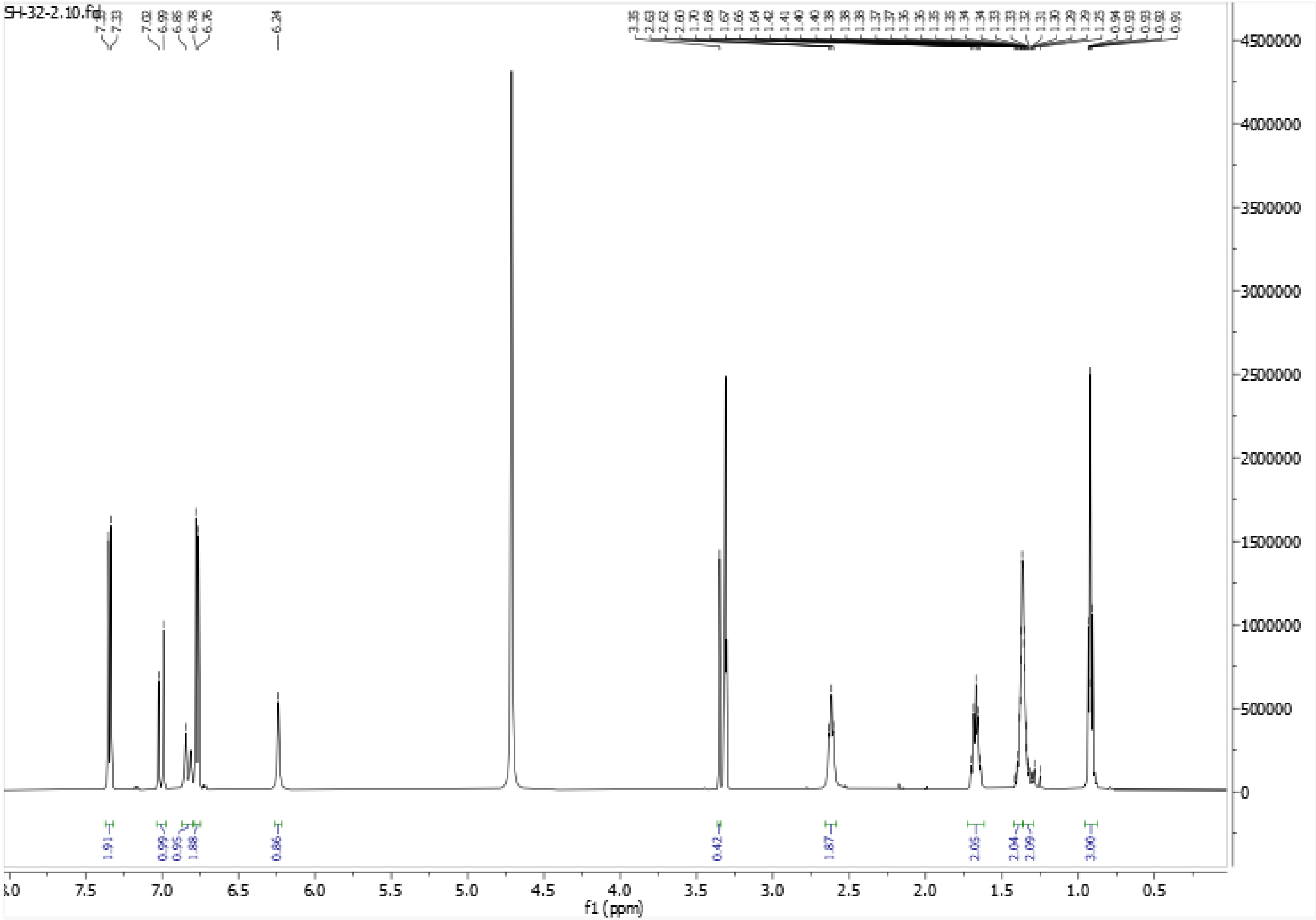

Figure S18. ^{13}C-NMR spectrum of compound **16**

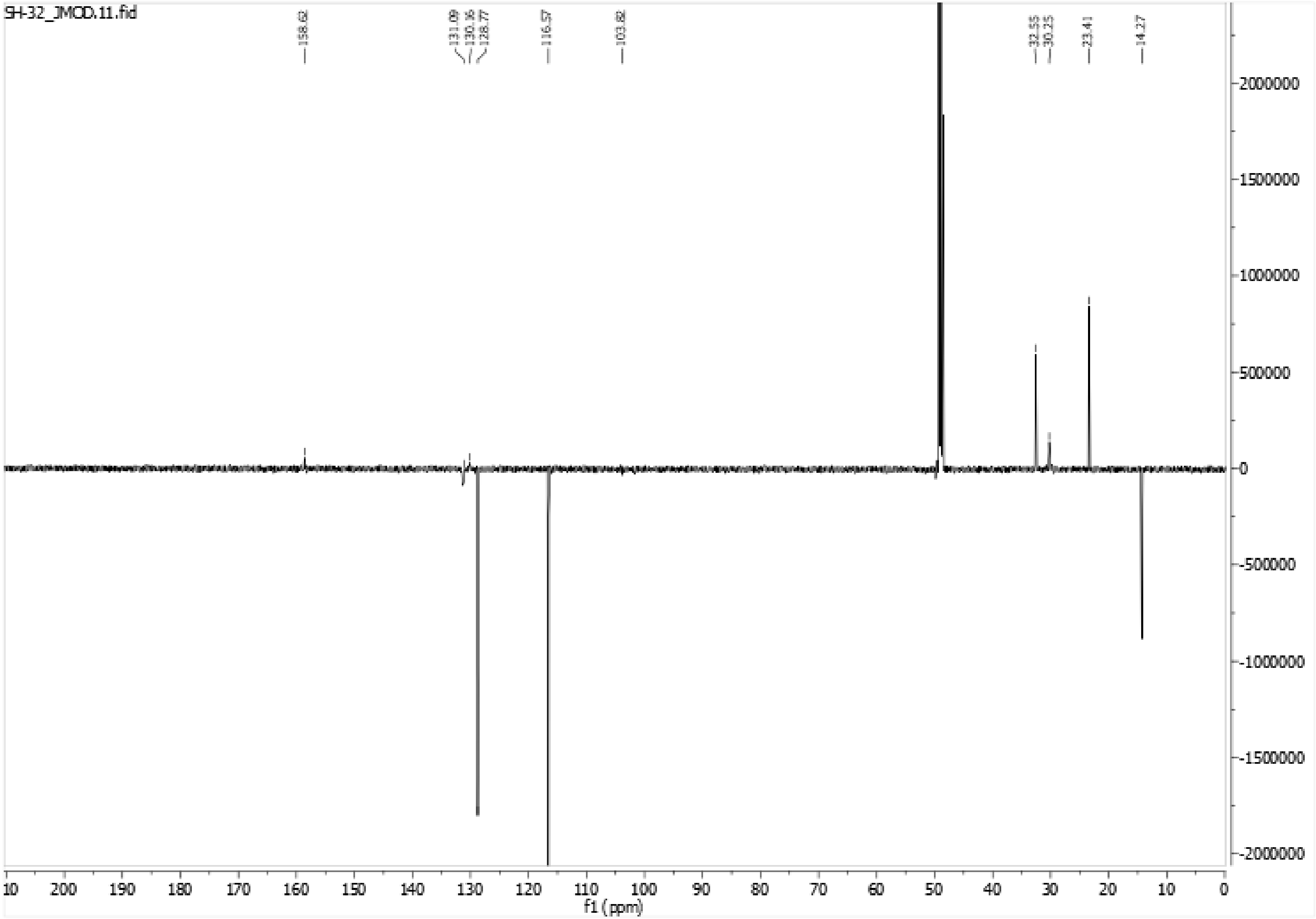

Figure S19. ^{1}H-NMR spectrum of compound **17**

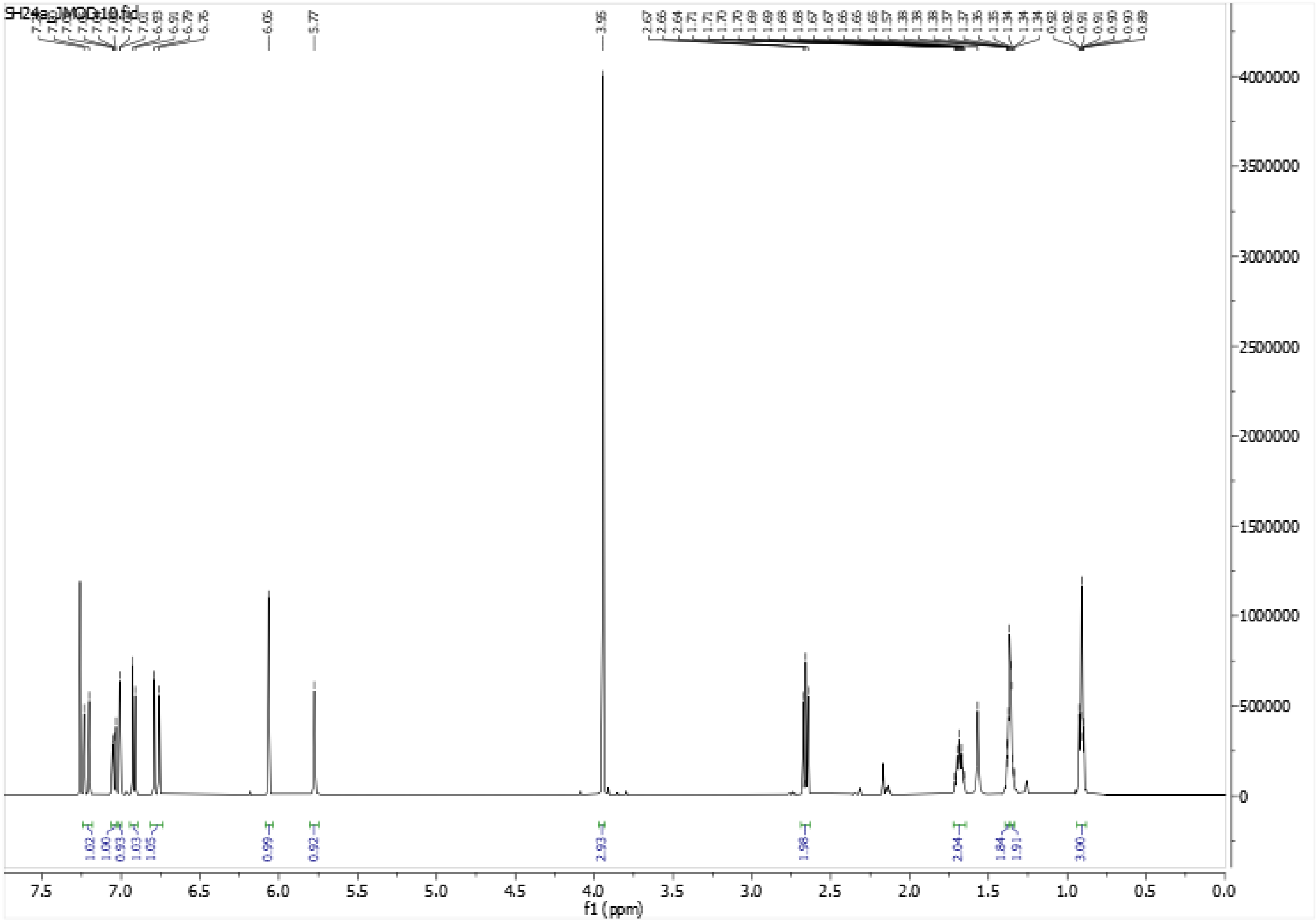

Figure S20. ^{13}C-NMR spectrum of compound **17**

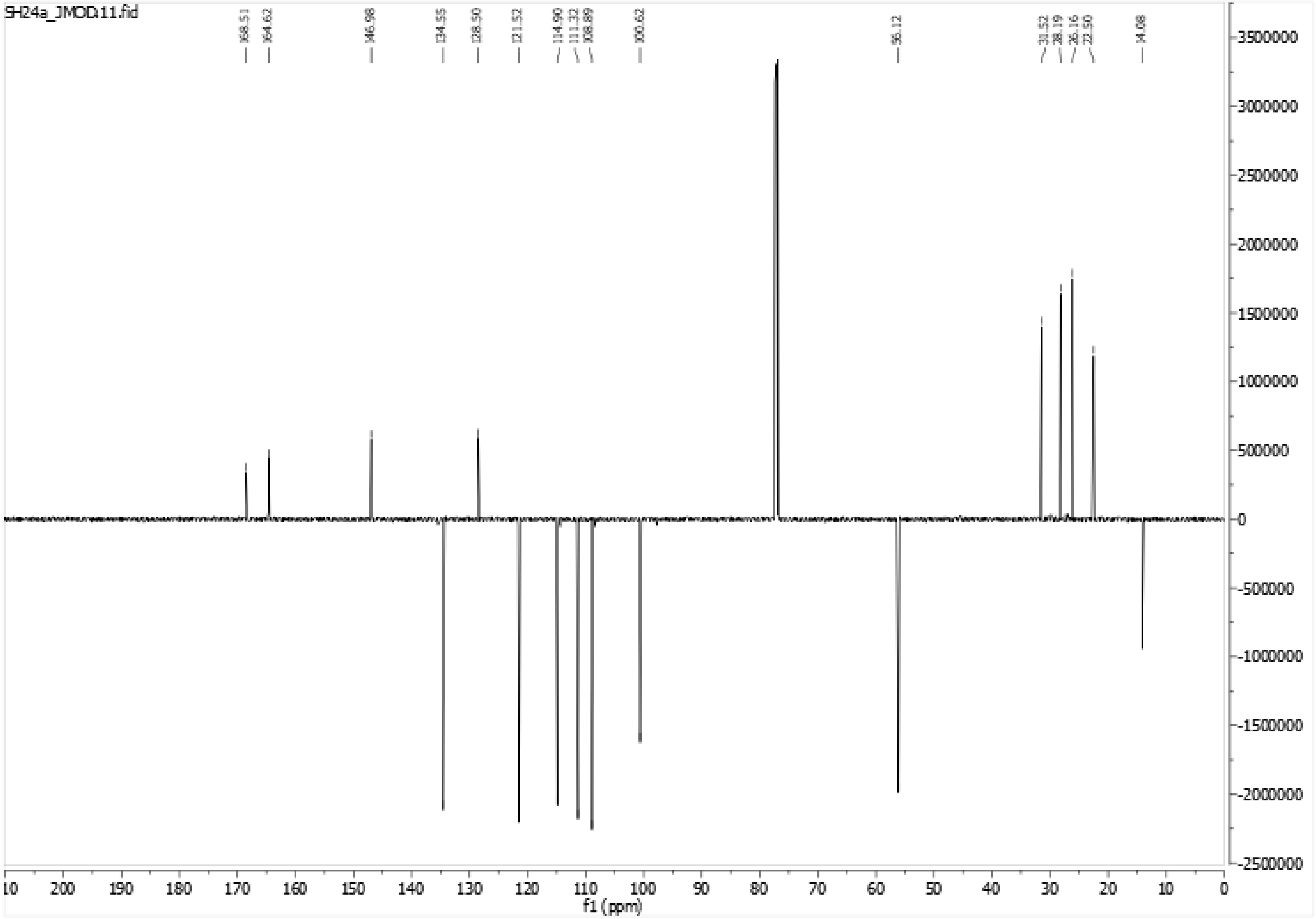

Figure S21. ^{1}H-NMR spectrum of compound **18**

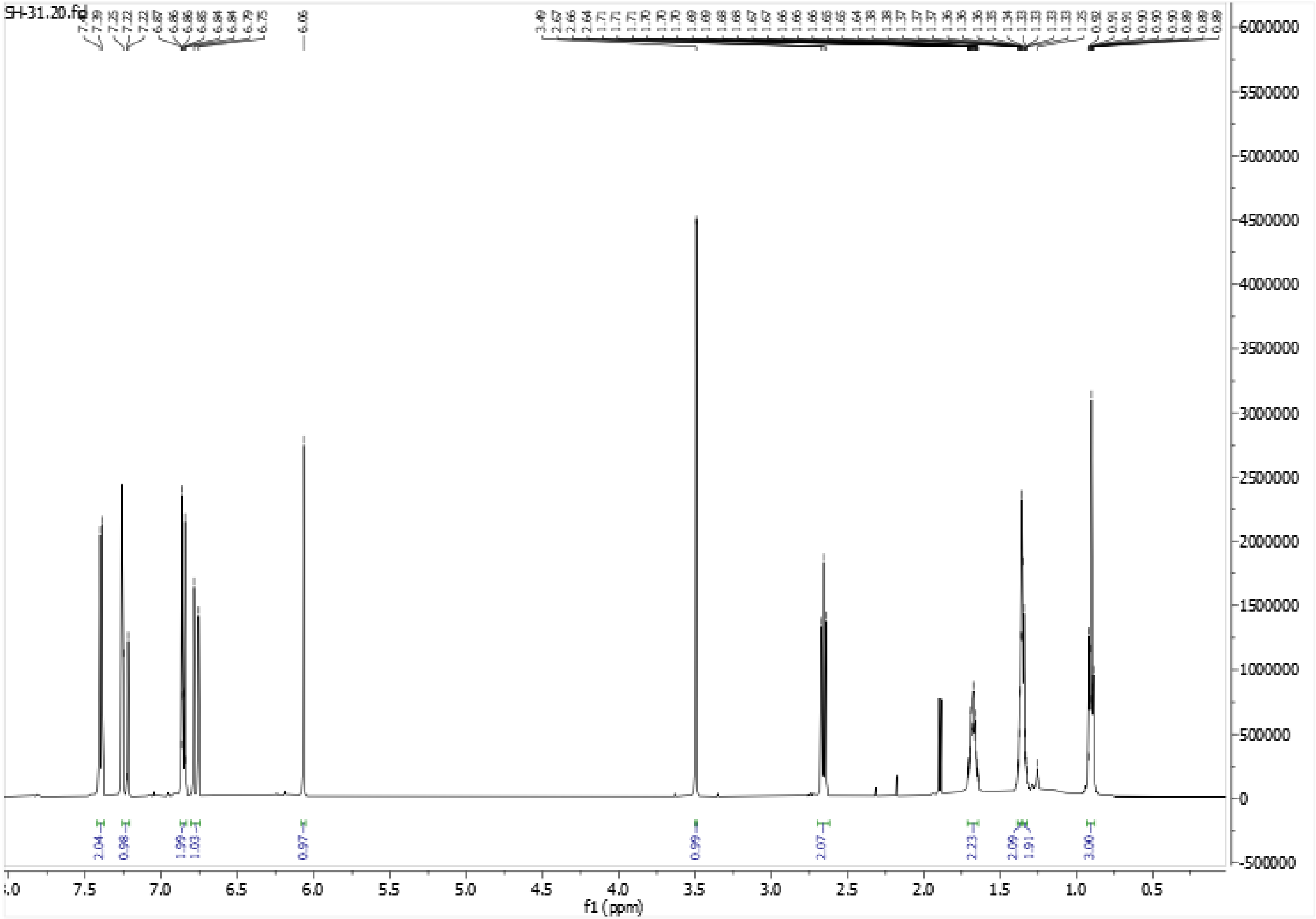

Figure S22. ^{13}C-NMR spectrum of compound **18**

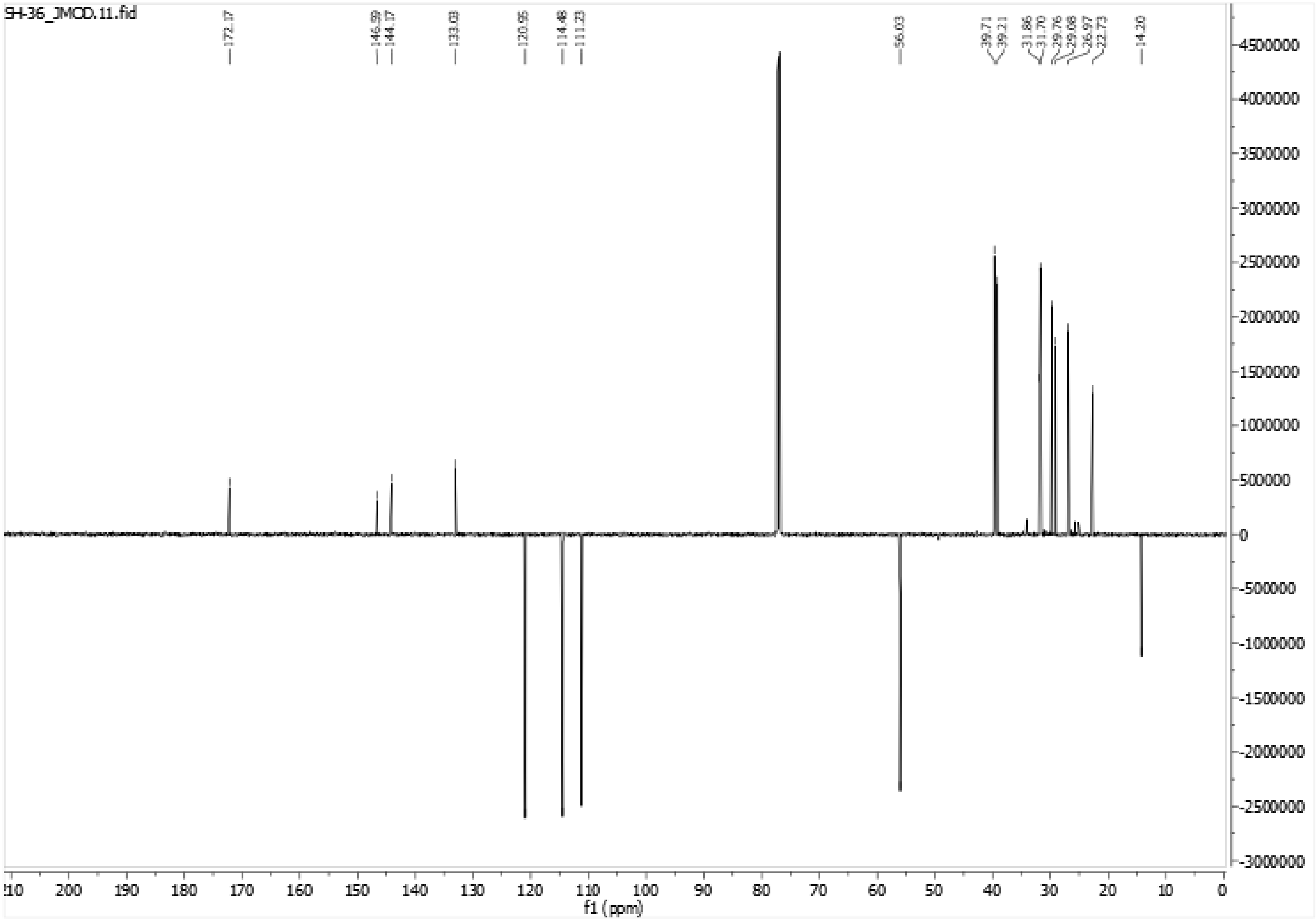

Figure S23. ^{1}H-NMR spectrum of compound **22**

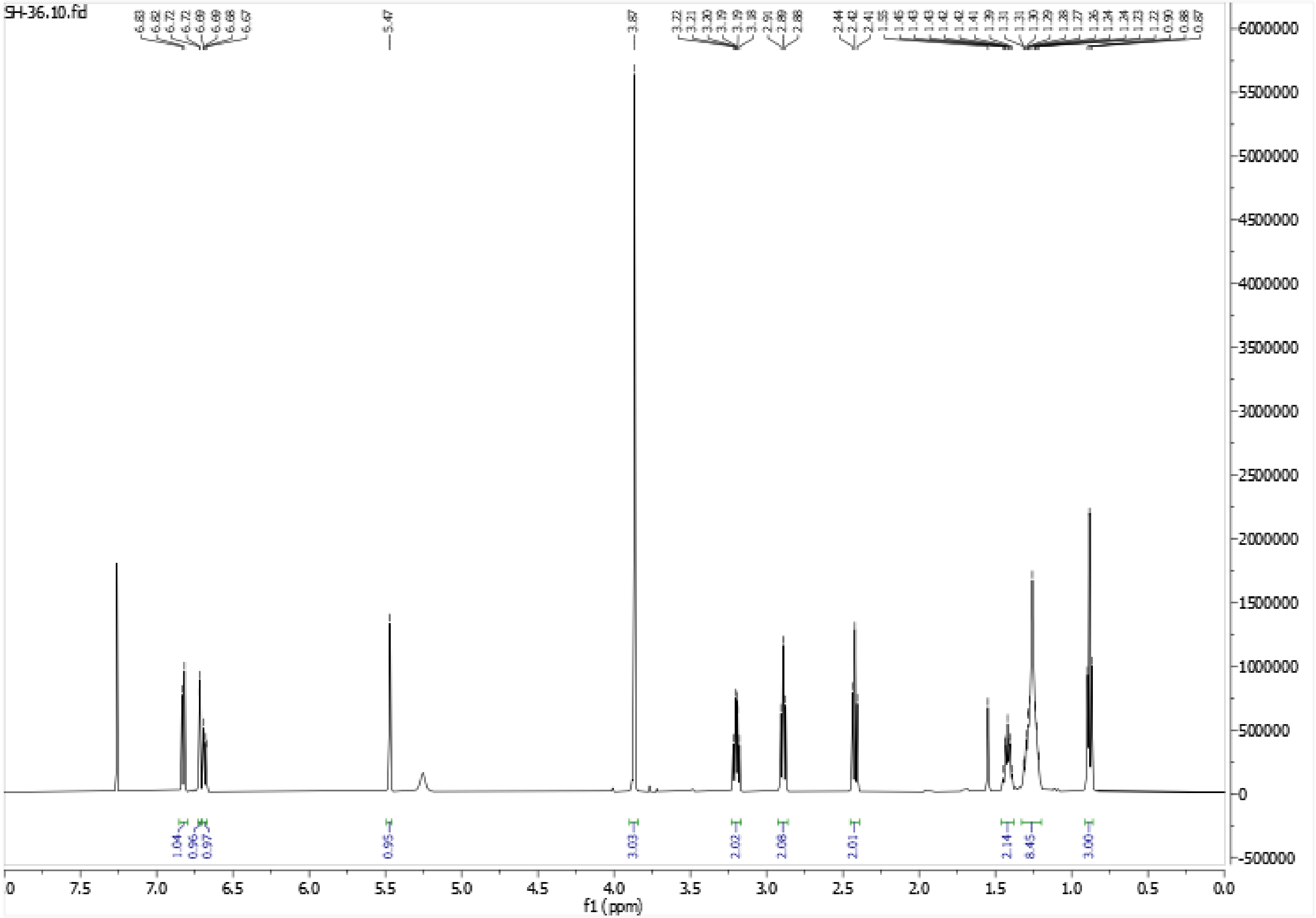

Figure S24. ^{13}C-NMR spectrum of compound **22**

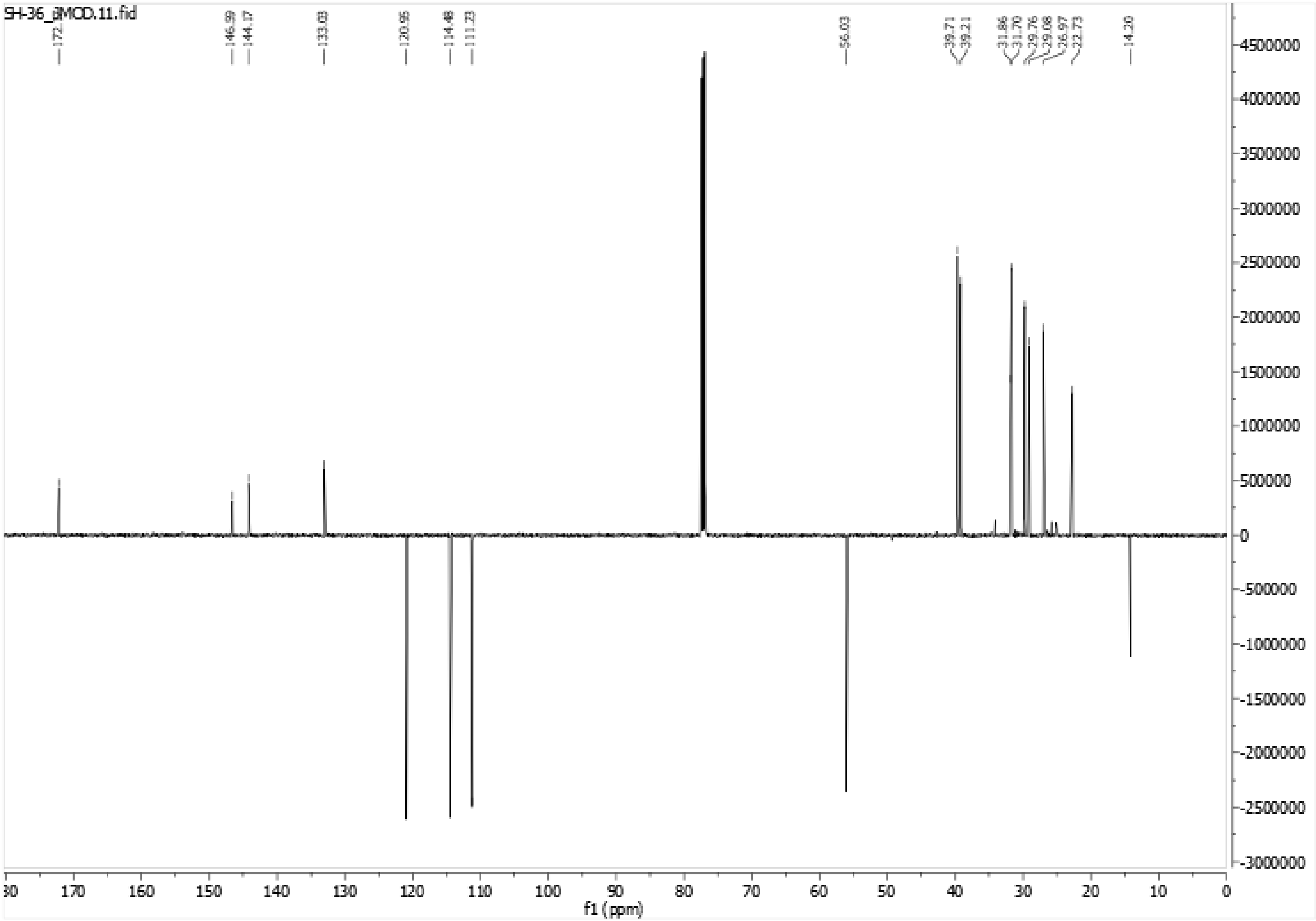

Figure S25. Correlation between the antiplatelet and COX-1 inhibitory activities of the tested compounds. Compounds inactive (IC_{50} > 100 µM on either bioassay, i.e., **12**, **15**, **18**, and **22**) are not included but they fit in the general trend.

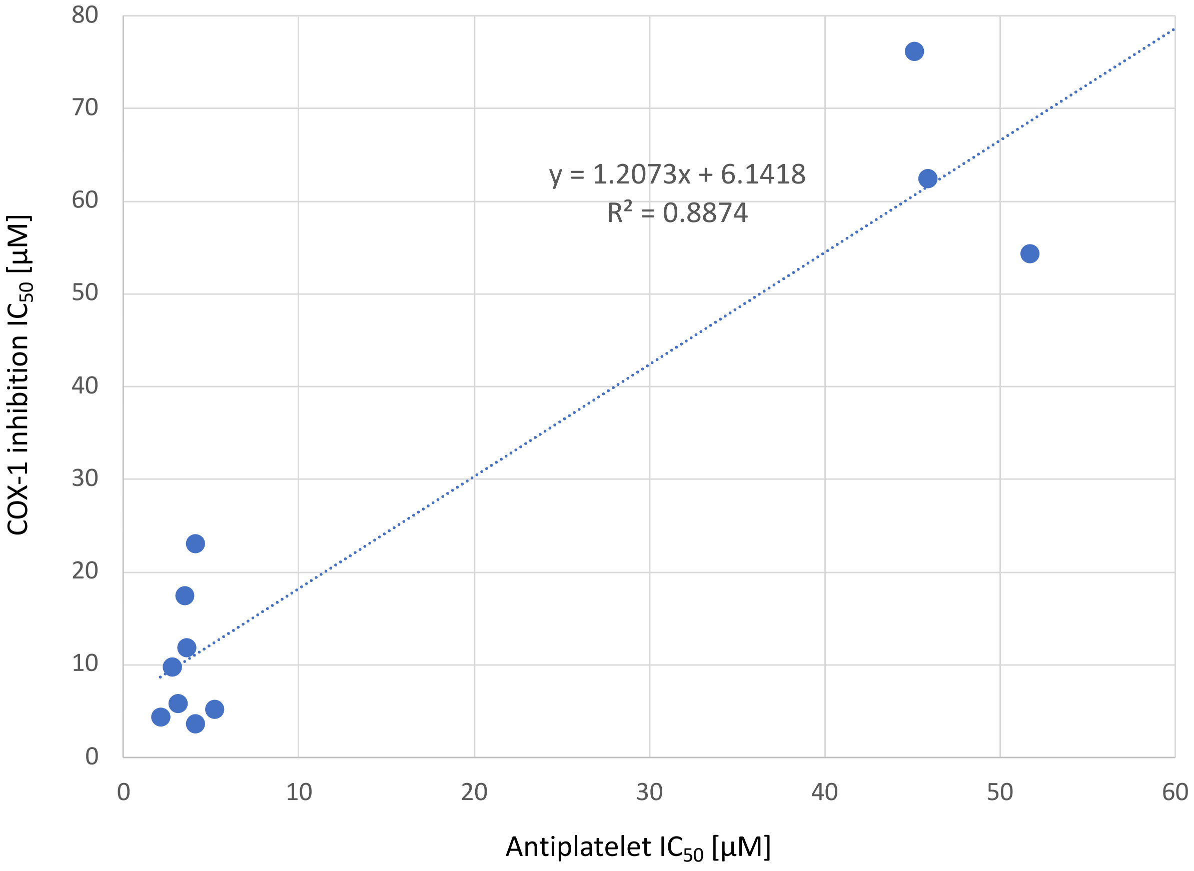

Table S1. Docking results obtained using AutoDockTools 1.5.7 and Discovery Studio visualizer (21.1.0.20298)

Compound	Binding energy (Kcal/mole)	Number of interacting AA	Number of conventional H-bonds	AA involved in H-bonds
**1**	-8.68	9	2	Val A:349, Ser A:530
**2**	-8.76	6	-	-
**3**	-8.50	9	1	Ile A:523
**4**	-8.38	11	1	Ser A:530
**5**	-8.01	11	1	Tyr A:355
**6**	-7.70	8	2	Val A:349, Ser A:530
**11A**	-9.29	9	-	-
**11B**	-8.93	9	1	Tyr A:385
**11C**	-8.21	4	-	-
**12A**	-9.23	6	3	Tyr A:385, Gly A:533, ASN A:375
**12B**	-8.76	5	1	GLY A:533
**12C**	-8.22	5	1	ASN A:375
**13A**	-8.30	7	1	Arg A:120
**13B**	-9.20	9	1	ASN A:375
**13C**	-8.85	11	-	-
**14**	-8.88	10	-	-
**15**	-8.36	9	1	Met A:522
**16**	-8.47	8	1	Gly A:533
**17**	-9.50	9	-	-
**18**	-9.32	12	1	Arg A:376
**22**	-8.54	10	2	Tyr A:385, Ile A:523
